# Association between the triacylglycerol–glucose index, TyG-BMI and hypertension in children and adolescents: a cross-sectional study with machine-learning-based risk stratification

**DOI:** 10.3389/fendo.2026.1964709

**Published:** 2026-10-16

**Authors:** Jialiang Zhu, Chen Zhang, Jialin Sun, Huiyu Du, Wenxin Zhao, Gang Tian, Jingge Zhao, Dandan Tian, Mengwei Wang, Zhanfeng Liu, Min Liu, Yibin Hao

**Affiliations:** 1 Department of Cardiology, Henan Provincial People’s Hospital, Zhengzhou University People’s Hospital, Zhengzhou, Henan, China; 2 Department of Hypertension, Henan Provincial People’s Hospital, Zhengzhou University People’s Hospital, Zhengzhou, Henan, China; 3 Clinical medicine, Henan University of Science and Technology, Luoyang, Henan, China; 4 Henan Province Hypertension Precision Prevention and Control Engineering Research Center, Henan Provincial People’s Hospital, Zhengzhou University People’s Hospital, Zhengzhou, Henan, China; 5 Department of Research and International Affairs, Zhengzhou University People’s Hospital, Henan Provincial People’s Hospital, Zhengzhou, Henan, China; 6 Henan Provincial Center for Disease Control and Prevention, Zhengzhou, China; 7 Henan Engineering Technology Research Center for Prevention and Treatment of Panvascular Diseases, Henan Provincial People’s Hospital, Zhengzhou University People’s Hospital, Zhengzhou, Henan, China

**Keywords:** children and adolescents, hypertension, machine learning, risk stratification, triacylglycerol–glucose index, TyG-BMI

## Abstract

**Background:**

Hypertension in children and adolescents is closely linked to obesity and metabolic disturbance. The triacylglycerol–glucose (TyG) index and TyG-BMI are surrogate markers of insulin resistance, but their associations with pediatric hypertension remain incompletely characterized.

**Methods:**

This cross-sectional study included 25,886 children and adolescents aged 7–17 years from 18 prefecture-level cities in Henan Province, China. The TyG index was calculated as ln[triglycerides (mg/dL) × fasting plasma glucose (mg/dL)/2] and TyG-BMI as TyG × BMI. Hypertension was defined as a blood pressure at or above the 95th percentile for age, sex and height (mean of the last two of three measurements). Logistic regression (Models I–IV) estimated odds ratios (ORs) per 1 SD and across quartiles, with non-linearity and effect modification assessed on both scales. Eleven machine-learning models were developed on a stratified 70:30 split, with imputation and feature selection performed strictly within the training set and discrimination (area under the receiver-operating characteristic curve, AUC) evaluated in the held-out validation set.

**Results:**

Among 25,886 participants, 1,798 (6.95%) had hypertension. Median TyG was 8.05 (IQR 7.77–8.35) and median TyG-BMI 154 (IQR 133–180). Each 1-SD increment in TyG was associated with hypertension in all models (Model IV 1.306 (95% CI 1.145–1.489)), and the association was graded across quartiles; TyG-BMI showed stronger associations (Model IV 2.109 (95% CI 1.985–2.241)). No multiplicative product term was significant after correction, whereas on the additive scale all six relative excess risk estimates were positive with confidence intervals excluding zero. In the held-out validation set, discrimination was moderate (best AUC 0.7544, 95% CI 0.7302–0.7777, for TyG and 0.7534, 0.7298–0.7759, for TyG-BMI), and adding either index to the same baseline model already containing BMI increased discrimination only marginally (about 0.003 in AUC).

**Conclusions:**

Higher TyG and TyG-BMI were associated with hypertension in Chinese children and adolescents, more strongly for TyG-BMI. No multiplicative interaction survived correction, but all additive-scale estimates were positive, so effect modification cannot be excluded. Machine-learning models built from routinely available variables showed only moderate discrimination and remain hypothesis-generating tools for risk stratification rather than validated prediction models. Longitudinal studies are needed to assess causality.

## Introduction

Hypertension in children and adolescents has emerged as a public health issue that can no longer be ignored. An updated systematic review and meta-analysis of 96 population-based studies, covering 443,914 children and adolescents across 21 countries, estimated the pooled global prevalence of childhood hypertension at 4.28% when hypertension was defined from repeated in-office measurements, at 6.67% when out-of-office measurements were also taken into account, and reported that the prevalence nearly doubled between 2000 and 2020, increasing from 3.40 to 6.53% in boys and from 3.02 to 5.82% in girls (1). Earlier systematic reviews and population-based analyses had likewise reported a rising prevalence in this age group, although the estimates varied considerably with the definition of hypertension and the number of measurements used (2, 3). The most recent Chinese guideline likewise identifies childhood hypertension as a priority for prevention, and recommends screening from the age of three years (4). This increase is closely linked to rising obesity rates, reduced physical activity, higher dietary sodium intake and urbanization. The current state of identification and management is nonetheless unsatisfactory: up to 90% of children do not receive regular blood pressure screening (5), and the value of universal screening in this age group remains a matter of debate, the US Preventive Services Task Force having concluded that the evidence is insufficient to weigh its benefits against its harms (6); more than three-quarters of children with hypertension remain undiagnosed, and even fewer receive adequate treatment. Childhood hypertension is closely associated with subclinical cardiovascular disease, including left ventricular hypertrophy, increased carotid intima-media thickness, elevated pulse wave velocity, atherosclerotic change, retinal microangiopathy and proteinuria (7). A long-term follow-up analysis of the International Childhood Cardiovascular Cohort Consortium, published in 2024, reported that childhood hypertension was associated with an approximately 2.3-fold higher risk of fatal or non-fatal cardiovascular events over more than 30 years (7). These findings underline the importance of identifying the metabolic and lifestyle correlates of hypertension early in life.

By integrating triglycerides and fasting plasma glucose — two analytes closely linked to insulin resistance — the triacylglycerol–glucose (TyG) index reflects the interaction between glucose and lipid metabolism and serves as a practical surrogate marker of insulin resistance (8). When insulin resistance develops, the suppressive effect of insulin on adipocyte lipolysis is attenuated, increasing the release of free fatty acids, which in turn promotes hepatic triglyceride synthesis; at the same time, insulin-mediated glucose uptake in muscle and adipose tissue is reduced, raising fasting plasma glucose (9). Combining the TyG index with body mass index (TyG-BMI) has been reported to improve the assessment of insulin resistance relative to TyG alone (10).

Previous studies have shown that the TyG index is associated with the prognosis of several diseases. It has been associated with all-cause and cardiovascular mortality in patients with cardiometabolic syndrome (11), with microvascular complications in Asian patients with type 2 diabetes (12), with recurrent adverse cardiovascular events after coronary surgery (13), and with stroke incidence and post-stroke cognitive impairment (14, 15). In children, evidence is only beginning to accumulate: an analysis of 12,087 children and adolescents aged 7–17 years from the 2017 China National Nutrition and Health Surveillance reported that TyG and the metabolic score for insulin resistance were associated with several hypertension phenotypes (16). Nevertheless, the number of pediatric studies remains small, most are limited to a single outcome definition, and few have examined whether the association differs across subgroups or whether the index improves risk stratification. As blood pressure regulatory mechanisms and metabolic profiles differ substantially between children and adults, and as the adult definition of hypertension and the thresholds at which it is treated (17) have no direct pediatric equivalent, associations established in adults cannot be assumed to apply to pediatric populations.

We therefore examined the associations of TyG and TyG-BMI with hypertension in a large population of Chinese children and adolescents, evaluated whether these associations are modified by central adiposity, uric acid and heart rate, and assessed whether machine-learning models built from routinely available variables can contribute to risk stratification. Because the study is cross-sectional, the analyses are explicitly framed as the characterization of associations and the generation of hypotheses rather than as causal inference.

## Methods

### Study design and participants

This cross-sectional survey was conducted between 2023 and 2024. The research team recruited a total of 28,844 students from primary, junior high and senior high schools across 18 prefecture-level cities in Henan Province, China, and collected baseline demographic, anthropometric, lifestyle and laboratory data. Participants were free of a diagnosis of hypertension at baseline and had complete data on the exposure variables (serum triglycerides and fasting plasma glucose) and on the outcome (at least two retained blood pressure records). Participants were excluded if they (1) had cognitive, mental, hearing or other related impairments; (2) had incomplete information on fasting plasma glucose, glycated hemoglobin, triglycerides or blood pressure; or (3) had a diagnosis of hypertension at baseline or only one retained blood pressure record. After these exclusions (n = 932; 853 with missing triglycerides or fasting plasma glucose and 79 with missing glycated hemoglobin; note that the 79 participants with missing glycated hemoglobin were excluded, not imputed), 27,912 participants remained. Because the reference values used to define the outcome are tabulated only for children and adolescents aged 7–17 years, the primary analysis was restricted to this age range: the 2,026 participants (7.26%) outside it — 656 younger than 7 years and 1,370 aged 18 years or older, for whom the diagnostic thresholds applied by the study centers cannot be verified from the de-identified dataset — were excluded, leaving 25,886 participants in the primary analysis (**Figure 1**). These participants were reintroduced in a pre-specified sensitivity analysis (**Supplementary Table S7**).

**Figure 1:**
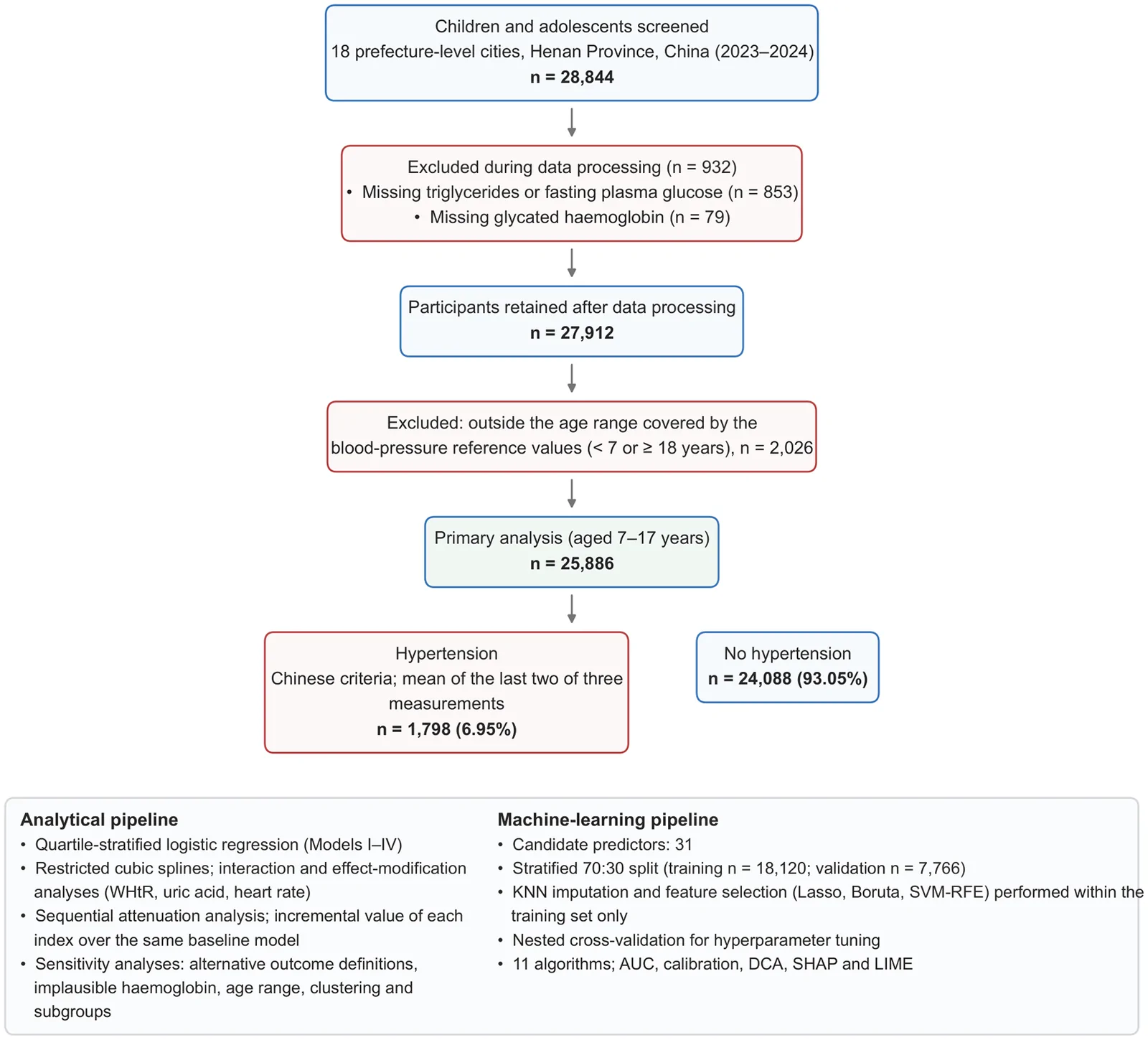
Study flow chart of participant selection and of the analytical pipelines. Participants were recruited from 18 prefecture-level cities in Henan Province, China, in 2023–2024; after the exclusions shown, 27,912 participants remained and 25,886 aged 7–17 years entered the primary analysis. The lower panels summarize the analytical pipeline and the machine-learning pipeline. AUC, area under the receiver-operating characteristic curve; DCA, decision curve analysis; LIME, local interpretable model-agnostic explanations; SHAP, Shapley additive explanations; SVM-RFE, support vector machine recursive feature elimination; WHtR, waist-to-height ratio.

Exposures were analyzed on both the continuous scale (per 1 SD) and the quartile scale. Quartiles of the triacylglycerol–glucose (TyG) index and of TyG-BMI were constructed within the analysis set, with the first quartile serving as the reference category in all regression models.

The study was approved by the Medical Ethics Committee of the Henan Provincial Center for Disease Control and Prevention (approval number 2022-KY-006-02). A revised informed consent form (V2.0/2022.07.10) was provided to all participating children and adolescents and their guardians; each participant and guardian signed the informed consent form and participated voluntarily. All methods comply with the relevant guidelines and regulations.

### Blood pressure measurement and outcome definitions

Blood pressure (systolic and diastolic, recorded in mmHg) was measured with an Omron HBP-1300 or HBP-1320 electronic monitor (Omron Healthcare, Kyoto, Japan), a validated oscillometric device with a display resolution of 1 mmHg; each monitor was supplied with three cuff sizes (S, M and L). All measurements were performed by trained investigators using the same device model at each site. The protocol required a quiet, separate room at an ambient temperature of 18–24 °C, no strenuous exercise or food and no caffeine-containing drink in the preceding hour, and at least five minutes of seated rest with the bladder emptied and the elbow resting on a table. The cuff was selected according to the mid-arm circumference and applied to the bare upper arm; the right arm was used throughout, and the left arm only when the right arm was affected by disease. To reduce the influence of the white-coat effect, blood pressure was measured three times on non-consecutive days at approximately the same time of day, and the participant’s blood pressure was taken as the mean of the last two of the three measurements (that is, the mean of the readings obtained on the second and third occasions); this averaging was applied before, and not after, comparison with the reference values, and participants with fewer than two retained measurements were excluded. The repeated measurements were used only to derive the hypertension indicator: the de-identified dataset available for the present analysis contains that derived indicator and not the individual readings, as noted in the Limitations.

The primary outcome, hypertension, was defined as a systolic or diastolic blood pressure (in mmHg) at or above the 95th percentile for the corresponding age, sex and height, according to the Chinese national reference values for children and adolescents aged 7–17 years (18); the 2024 revision of the Chinese hypertension guideline applies the same percentile-based definition in this age group (4), and the corresponding European guideline uses the same 95th-percentile principle with a different reference population (19). Because the reference values are tabulated only for ages 7–17 years, the primary analysis was restricted to this range and no extrapolated thresholds were used. Because the choice of reference population and of the averaging rule directly determines the observed prevalence, two alternative definitions were pre-specified for sensitivity analysis: (i) the Chinese reference applied to the mean of all three measurements, and (ii) the European reference applied to the mean of all three measurements. The three definitions yielded prevalences of 6.95%, 8.30% and 5.27%, respectively (**Supplementary Table S1**).

For the 2,026 participants outside the reference range, the diagnostic thresholds used by the study centers cannot be verified, because the de-identified dataset available for this analysis contains the derived hypertension indicator but no blood-pressure values. These participants were therefore excluded from the primary analysis rather than classified with an extrapolated threshold. In a pre-specified sensitivity analysis that re-included all 27,912 participants of the original analysis set, the estimates were unchanged (Model IV odds ratio per 1 SD 1.347 (95% CI 1.186–1.529) for TyG and 2.087 (95% CI 1.967–2.215) for TyG-BMI; **Supplementary Table S7**).

### Exposure variables

Venous blood samples were collected after an overnight fast. Serum triglycerides, total cholesterol, low-density lipoprotein cholesterol, high-density lipoprotein cholesterol, fasting plasma glucose, glycated hemoglobin, total protein, serum creatinine, uric acid and hemoglobin were measured on an automated biochemical analyzer. Triglycerides and fasting plasma glucose were reported in mmol/L. The TyG index was calculated as:


$$TyG=ln\left[TG\left(mg/dL\right)\times FPG\left(mg/dL\right)/2\right]$$

where TG (mg/dL) = TG (mmol/L) × 88.57 and FPG (mg/dL) = FPG (mmol/L) × 18.0. TyG-BMI was calculated as TyG × BMI, where BMI was weight (kg) divided by the square of height (m) (20).

We verified the recalculation of the exposure. The TyG index was recomputed independently from the raw triglyceride and fasting plasma glucose concentrations (both in mmol/L) with the published conversion factors 88.57 and 18.0; the recomputed values agreed with the exposure variable in the analytical dataset for all 25,886 participants of the analysis set, with a maximum absolute difference of 3.6 × 10⁻¹^5^ (relative difference 3.9 × 10⁻¹^6^, that is, floating-point precision). TyG-BMI, recomputed as TyG × BMI, agreed with the corresponding variable to within 1.1 × 10⁻¹³. The verification is reproduced in the analysis code (**Supplementary Material 2**). Exposure variables were used on both the continuous (per 1 SD) and the quartile scale.

### Covariates

A total of 31 candidate predictors were collected: age; heart rate; low-density lipoprotein cholesterol; high-density lipoprotein cholesterol; total cholesterol; total protein; serum creatinine; uric acid; hemoglobin; BMI; a body shape index (ABSI); corpulence index; waist-to-height ratio (WHtR); moderate-to-vigorous physical activity (MVPA); median physical exercise time; walking volume; workday night sleep duration; weekend night sleep duration; workday sedentary time; weekend sedentary time; sex; snoring frequency; smoking; alcohol use; milk tea consumption; asthma; education level; paternal hypertension; maternal hypertension; paternal grandparental hypertension; and maternal grandparental hypertension. Four models were fitted: Model I was unadjusted; Model II was adjusted for age, sex, smoking, alcohol use and milk tea consumption; Model III additionally included heart rate, low- and high-density lipoprotein cholesterol, total cholesterol, total protein, serum creatinine, uric acid and hemoglobin; and Model IV further included workday and weekend sleep duration, workday and weekend sedentary time, MVPA, physical exercise, walking volume, snoring and family history of hypertension in parents and grandparents.

### Statistical analysis

Continuous variables are presented as medians with interquartile ranges and were compared using the Mann–Whitney U test; categorical variables are presented as frequencies and percentages and were compared using the χ² test. Missing values were rare and were handled by median imputation for the descriptive and regression analyses: hemoglobin (83 implausible zero values, 0.30%, set to missing before imputation) and heart rate (2 values, 0.01%). Glycated hemoglobin was not imputed: the 79 participants (0.28%) with a missing value were excluded before analysis (**Figure 1**). Multivariable logistic regression was used to estimate ORs and 95% confidence intervals (CIs) for hypertension per 1-SD increment in the exposure and across the pre-specified exposure quartiles, with a test for linear trend across quartile categories.

Restricted cubic splines were fitted with three to seven knots placed at recommended percentiles; the number of knots was selected by the Akaike information criterion (AIC), and the model with the lowest AIC was retained (**Supplementary Table S2**). Departure from linearity was tested by a likelihood ratio test comparing the spline model with a model containing only a linear term; the reference was set at the 10th percentile of the exposure, and the fitted curves were inspected for evidence of an internal minimum.

Because the data are cross-sectional, no mediation analysis was performed. Instead, effect modification was assessed in two ways. First, participants were stratified at the median of WHtR and of uric acid, and the exposure–outcome association was estimated within each stratum. Second, product terms between the standardized exposure and the standardized modifier were added to the fully adjusted model. Both the exposure and the modifier were centered and scaled to 1 SD before the product term was formed, so that all contrasts are expressed per 1 SD, and the reference category of the interaction model is a participant at the mean of both the exposure and the modifier. Interaction was tested on the multiplicative scale by a likelihood ratio test comparing the models with and without the product term. On the additive scale, RERI was calculated from the coefficients of the same continuous product model as exp(β1 + β2 + β12) − exp(β1) − exp(β2) + 1, with 95% CIs obtained by the delta method (21); this is the continuous per-1-SD analog of the conventional four-category RERI and assumes log-linearity of the exposure–outcome odds ratio and multiplicativity of the modifier on the odds scale. Because the RERI and the multiplicative test are derived from the same model, the two scales are not independent, and a divergence between them reflects the non-collapsibility of the odds ratio rather than a genuine contradiction. All six interaction tests (three modifiers × two panels) were additionally evaluated against a Bonferroni-corrected significance level of 0.0083. The same procedure was applied to heart rate. In addition, a sequential attenuation analysis was performed, in which WHtR and BMI were added stepwise to the fully adjusted model (**Supplementary Table S8**); this analysis is descriptive and does not estimate a causal mediation effect.

Cluster-level identifiers (school or city) were not available in the de-identified dataset, so the clustering of participants within schools and cities could not be modelled directly. Instead, the consequences of ignoring clustering were quantified analytically through the design effect DE = 1 + (m − 1)ρ, where m is the mean cluster size and ρ the intra-cluster correlation. Standard errors were inflated by √DE over a grid of mean cluster sizes (30–400) and intra-cluster correlations (0.01–0.05); these values span the range reported for school-based surveys of blood pressure, in which intra-cluster correlations of 0.01–0.05 and cluster sizes of 30–200 are typical (**Supplementary Table S9**).

Five sensitivity analyses were performed: the two alternative outcome definitions described above; re-estimation of all models after excluding participants with implausible hemoglobin values; re-inclusion of the participants outside the reference age range (**Supplementary Table S7**); the analytic cluster-sensitivity assessment described above (**Supplementary Table S9**); and subgroup analyses by sex and by heart-rate category, defined at 71 beats per minute (the 10th percentile of the observed distribution is 71.5 beats per minute). All tests were two-sided and a p value below 0.05 was considered statistically significant unless stated otherwise. Analyses were performed in Python 3.11 with pandas, NumPy, statsmodels, scikit-learn, XGBoost and LightGBM.

The complete analysis code, comprising the scripts that regenerate every table and figure from the analysis dataset, is provided as **Supplementary Material 2** (analysis code). In addition, the exposure was independently recalculated from the raw triglyceride and fasting plasma glucose concentrations with the published conversion factors, and the results of that verification are reported below.

### Feature selection and machine-learning models

Two parallel panels were constructed: a TyG panel and a TyG-BMI panel, each containing the 31 candidate predictors together with the corresponding exposure (32 variables in total). The dataset was split into a training set (70%, n = 18,120, 1,259 with hypertension) and a held-out validation set (30%, n = 7,766, 539 with hypertension) using stratified random sampling with a fixed random seed. To avoid information leakage, all data-dependent steps were performed on the training set only (22, 23): missing values were imputed with k-nearest-neighbor imputation (k = 5) fitted on the training set, and three feature-selection algorithms were applied within the training set using five-fold cross-validation. The Lasso algorithm performs variable selection through L1 regularization and is well suited to correlated predictors (24); Boruta compares the importance of each feature with that of randomly permuted shadow features and can capture non-linear associations (25); and recursive feature elimination with a support vector machine (SVM-RFE) ranks features by their influence on the classification hyperplane (26). The final predictor set for each panel was defined by a pre-specified two-step rule: features selected by all three algorithms were retained, and because this strict intersection contained fewer than four features in both panels (two in the TyG panel and one in the TyG-BMI panel), the rule fell back to the eight features selected most frequently across the three algorithms. Neither exposure was retained by all three algorithms.

Eleven algorithms were evaluated: logistic regression, decision tree, Lasso, ridge classifier, elastic net, k-nearest neighbors, random forest, extreme gradient boosting (XGBoost), support vector machine, LightGBM and a multilayer perceptron. Hyperparameters were tuned by grid search within an inner three-fold cross-validation loop nested inside a five-fold outer loop of the training set, so that performance estimates were not optimistically biased (23). The final models were refitted on the complete training set with the selected hyperparameters and evaluated once in the held-out validation set. The ridge classifier and the support vector machine, which do not produce probabilities directly, were wrapped in Platt scaling (five-fold sigmoid calibration) so that the Brier score and the calibration curves of all eleven algorithms were on a comparable probability scale. Because the outcome is imbalanced (approximately 7%), a default probability threshold of 0.50 classifies almost every participant as unaffected. Classification metrics (sensitivity, specificity, positive and negative predictive value and F1) are therefore reported both at the default threshold of 0.50 and at the Youden-optimal threshold, which maximizes sensitivity + specificity − 1 in the validation set, the numeric value of the threshold used for each of the eleven models being reported in **Supplementary Table S6**. Because that threshold is selected in the same set in which the classification metrics are then calculated, all threshold-dependent metrics (accuracy, sensitivity, specificity, positive and negative predictive value and F1) are reported as exploratory; discrimination and calibration, which are threshold-independent, are the primary measures of performance. The consequences of the low sensitivity obtained at the default threshold are addressed explicitly in the Discussion. Discrimination was assessed by the AUC with 95% CIs obtained from 500 bootstrap resamples, and the AUCs of TyG and TyG-BMI were compared with the DeLong test for correlated curves. Calibration was assessed with calibration curves and the Brier score, and clinical usefulness with decision curve analysis (DCA) across threshold probabilities from 2 to 60%. Reporting follows the TRIPOD+AI statement (27), and the completed checklist is provided as **Supplementary Material 1**.

Model interpretation used SHapley Additive exPlanations (SHAP) and Local Interpretable Model-agnostic Explanations (LIME). SHAP values were computed with a tree explainer for the best-performing tree-based model in each panel; global importance was summarized as the mean absolute SHAP value, and feature dependence was visualized with dependence plots. LIME explanations decomposed the prediction for individual participants.

## Results

### Baseline characteristics

Of the 25,886 participants in the primary analysis, 1,798 (6.95%) had hypertension according to the primary definition (**Figure 1**). Median age was 12.5 years (IQR 9.8–15.2) and 13,353 (51.6%) were male. Baseline characteristics according to hypertension status are shown in **Table 1**. Participants with hypertension were older, had a higher heart rate, higher low-density lipoprotein cholesterol, total cholesterol, total protein, serum creatinine, uric acid and hemoglobin, a higher BMI, ABSI and WHtR, and lower high-density lipoprotein cholesterol; they were more frequently male, reported snoring more often, slept less on workdays and spent more time sedentary. Median TyG was 8.05 (IQR 7.77–8.35) overall and was higher in participants with hypertension than in those without (8.23, IQR 7.93–8.58 versus 8.03, IQR 7.75–8.33; p < 0.001). Median TyG-BMI was 154 (IQR 133–180) overall and was also higher in participants with hypertension (186, IQR 155–230 versus 152, IQR 132–177; p < 0.001).

**Table 1:** Baseline characteristics of the study population according to hypertension status.

Characteristic	Overall (N = 25,886)	No hypertension (N = 24,088)	Hypertension (N = 1,798)	p value
Age (years)	12.53 (9.78, 15.24)	12.48 (9.75, 15.18)	13.57 (10.39, 16.12)	<0.001
Heart rate (beats/min)	88.0 (79.0, 97.0)	87.5 (79.0, 96.5)	90.5 (80.5, 100.5)	<0.001
LDLC (mmol/L)	1.85 (1.52, 2.23)	1.84 (1.52, 2.21)	1.95 (1.61, 2.39)	<0.001
HDLC (mmol/L)	1.54 (1.33, 1.78)	1.55 (1.34, 1.79)	1.41 (1.21, 1.64)	<0.001
TC (mmol/L)	3.82 (3.40, 4.27)	3.81 (3.40, 4.26)	3.90 (3.46, 4.39)	<0.001
Total protein (g/L)	75.9 (73.0, 78.9)	75.8 (72.9, 78.8)	77.1 (74.2, 80.1)	<0.001
SCre (μmol/L)	49.0 (42.0, 59.0)	49.0 (42.0, 58.0)	52.0 (43.0, 65.0)	<0.001
UA (μmol/L)	307.0 (260.0, 367.0)	304.0 (258.0, 363.0)	349.0 (289.0, 422.0)	<0.001
Hb (g/L)	137.0 (130.0, 145.0)	136.0 (129.0, 145.0)	142.0 (133.0, 152.0)	<0.001
BMI (kg/m²)	19.1 (16.7, 22.0)	18.9 (16.6, 21.7)	22.6 (19.0, 27.2)	<0.001
ABSI	11.0 (10.2, 11.9)	11.0 (10.2, 11.8)	11.5 (10.6, 12.3)	<0.001
Corpulence index	1.9 (1.5, 2.7)	1.9 (1.5, 2.8)	1.5 (1.2, 2.2)	<0.001
WHtR	0.42 (0.39, 0.46)	0.41 (0.39, 0.45)	0.46 (0.41, 0.54)	<0.001
MVPA (min/week)	105 (40, 200)	105 (40, 200)	100 (40, 200)	0.684
Physical exercise (min/week)	210 (77, 400)	210 (77, 400)	201 (75, 400)	0.363
Walk (m/day)	30 (0, 100)	30 (0, 100)	30 (0, 100)	0.983
Workday night sleep (h/day)	8.83 (7.42, 9.67)	8.83 (7.42, 9.67)	8.33 (7.08, 9.50)	<0.001
Weekend night sleep (h/day)	10.00 (9.00, 10.58)	10.00 (9.00, 10.67)	9.92 (9.00, 10.50)	<0.001
Workday sedentary (h/day)	8.50 (7.00, 10.92)	8.50 (7.00, 10.83)	9.00 (7.33, 11.33)	<0.001
Weekend sedentary (h/day)	5.63 (3.50, 8.50)	5.50 (3.50, 8.50)	6.00 (3.52, 9.00)	0.005
TyG	8.05 (7.77, 8.35)	8.03 (7.75, 8.33)	8.23 (7.93, 8.58)	<0.001
TyG-BMI	154 (133, 180)	152 (132, 177)	186 (155, 230)	<0.001
Gender, n (%)				<0.001
Male	13353 (51.6%)	12283 (51.0%)	1070 (59.5%)	
Female	12533 (48.4%)	11805 (49.0%)	728 (40.5%)	
Snoring, n (%)				<0.001
At least 3 days a week	669 (2.6%)	582 (2.4%)	87 (4.8%)	
1-2 days per week	1833 (7.1%)	1648 (6.8%)	185 (10.3%)	
No	23384 (90.3%)	21858 (90.7%)	1526 (84.9%)	
Smoke, n (%)				0.002
Yes	339 (1.3%)	314 (1.3%)	25 (1.4%)	
Quit smoking	560 (2.2%)	500 (2.1%)	60 (3.3%)	
No	24987 (96.5%)	23274 (96.6%)	1713 (95.3%)	
Alcohol, n (%)				0.002
Yes	1170 (4.5%)	1062 (4.4%)	108 (6.0%)	
Quit drinking	3438 (13.3%)	3180 (13.2%)	258 (14.3%)	
No	21278 (82.2%)	19846 (82.4%)	1432 (79.6%)	
Milk tea, n (%)				0.056
Yes	10668 (41.2%)	9966 (41.4%)	702 (39.0%)	
No	15218 (58.8%)	14122 (58.6%)	1096 (61.0%)	
Asthma, n (%)				0.698
Yes	165 (0.6%)	153 (0.6%)	12 (0.7%)	
No	23902 (92.3%)	22251 (92.4%)	1651 (91.8%)	
Don't know	1819 (7.0%)	1684 (7.0%)	135 (7.5%)	
Education, n (%)				<0.001
Other / not specified	506 (2.0%)	445 (1.8%)	61 (3.4%)	
No formal school education	461 (1.8%)	419 (1.7%)	42 (2.3%)	
Incomplete primary school	1543 (6.0%)	1443 (6.0%)	100 (5.6%)	
Primary school graduation	2890 (11.2%)	2684 (11.1%)	206 (11.5%)	
Junior high school graduation	11096 (42.9%)	10314 (42.8%)	782 (43.5%)	
High school graduation	5506 (21.3%)	5125 (21.3%)	381 (21.2%)	
College graduate	2320 (9.0%)	2180 (9.1%)	140 (7.8%)	
Bachelor degree	1490 (5.8%)	1411 (5.9%)	79 (4.4%)	
Graduate students and above	74 (0.3%)	67 (0.3%)	7 (0.4%)	
Father hypertension, n (%)				0.505
Yes	722 (2.8%)	665 (2.8%)	57 (3.2%)	
No	23003 (88.9%)	21418 (88.9%)	1585 (88.2%)	
Don't know	2161 (8.3%)	2005 (8.3%)	156 (8.7%)	
Mother hypertension, n (%)				0.008
Yes	513 (2.0%)	460 (1.9%)	53 (2.9%)	
No	23212 (89.7%)	21623 (89.8%)	1589 (88.4%)	
Don't know	2161 (8.3%)	2005 (8.3%)	156 (8.7%)	
Paternal grandparents hypertension, n (%)				0.502
Yes	1230 (4.8%)	1154 (4.8%)	76 (4.2%)	
No	22495 (86.9%)	20929 (86.9%)	1566 (87.1%)	
Don't know	2161 (8.3%)	2005 (8.3%)	156 (8.7%)	
Maternal grandparents hypertension, n (%)				0.134
Yes	505 (2.0%)	481 (2.0%)	24 (1.3%)	
No	23220 (89.7%)	21602 (89.7%)	1618 (90.0%)	
Don't know	2161 (8.3%)	2005 (8.3%)	156 (8.7%)	

Continuous variables are presented as median (interquartile range) and compared with the Mann–Whitney U test; categorical variables are presented as n (%) and compared with the χ² test. Hypertension was defined using the Chinese reference values and the mean of the last two of three blood pressure measurements. ABSI, a body shape index; Hb, haemoglobin; HDLC, high-density lipoprotein cholesterol; HR, heart rate; LDLC, low-density lipoprotein cholesterol; MVPA, moderate-to-vigorous physical activity; SCre, serum creatinine; TC, total cholesterol; TyG, triacylglycerol–glucose index; UA, uric acid; WHtR, waist-to-height ratio.

### Associations of TyG and TyG-BMI with hypertension

Each 1-SD increment in TyG (1 SD = 0.4387) was associated with hypertension in the unadjusted model (Model I 1.557 (95% CI 1.488–1.630)) and the association persisted after adjustment for demographic and lifestyle factors (Model II 1.537 (95% CI 1.468–1.610)) and for biochemical covariates (Model III 1.298 (95% CI 1.139–1.480)) as well as after further adjustment for sleep, sedentary behavior, physical activity, snoring and family history (Model IV 1.306 (95% CI 1.145–1.489)). The association was graded across quartiles: relative to the first quartile, the ORs in the fourth quartile were 1.45 (95% CI 1.17–1.80) in Model III and 1.45 (95% CI 1.17–1.81) in Model IV (p for trend < 0.001) (**Table 2**).

**Table 2:** Associations of the TyG index with hypertension in children and adolescents.

Model	Contrast	OR (95% CI)	p value
Model I	Per 1 SD	1.557 (1.488–1.630)	<0.001
	Q2 vs Q1	1.39 (1.18–1.64)	<0.001
	Q3 vs Q1	1.70 (1.45–1.99)	<0.001
	Q4 vs Q1	3.05 (2.64–3.53)	<0.001
	p for trend		<0.001
Model II	Per 1 SD	1.537 (1.468–1.610)	<0.001
	Q2 vs Q1	1.38 (1.17–1.62)	<0.001
	Q3 vs Q1	1.67 (1.43–1.96)	<0.001
	Q4 vs Q1	2.97 (2.57–3.45)	<0.001
	p for trend		<0.001
Model III	Per 1 SD	1.298 (1.139–1.480)	<0.001
	Q2 vs Q1	1.14 (0.96–1.34)	0.139
	Q3 vs Q1	1.17 (0.99–1.40)	0.069
	Q4 vs Q1	1.45 (1.17–1.80)	<0.001
	p for trend		0.002
Model IV	Per 1 SD	1.306 (1.145–1.489)	<0.001
	Q2 vs Q1	1.14 (0.96–1.35)	0.132
	Q3 vs Q1	1.18 (0.99–1.40)	0.063
	Q4 vs Q1	1.45 (1.17–1.81)	<0.001
	p for trend		0.002

OR, odds ratio; CI, confidence interval. Model I, unadjusted; Model II, adjusted for age, sex, smoking, alcohol use and milk tea consumption; Model III, Model II plus heart rate, low- and high-density lipoprotein cholesterol, total cholesterol, total protein, serum creatinine, uric acid and haemoglobin; Model IV, Model III plus workday and weekend sleep duration, workday and weekend sedentary time, moderate-to-vigorous physical activity, physical exercise, walking volume, snoring and family history of hypertension. Quartiles were defined on the corrected exposure distribution. Hypertension was defined using the Chinese reference values and the mean of the last two of three measurements.

TyG-BMI showed stronger associations than TyG at every level of adjustment. Each 1-SD increment in TyG-BMI (1 SD = 38.45) was associated with hypertension with ORs of 2.071 (95% CI 1.989–2.157) (Model I), 2.121 (95% CI 2.031–2.214) (Model II), 2.108 (95% CI 1.985–2.238) (Model III) and 2.109 (95% CI 1.985–2.241) (Model IV). Across quartiles, the fourth quartile was associated with a more than threefold higher odds of hypertension relative to the first quartile (3.74 (95% CI 3.08–4.55) in Model III; 3.79 (95% CI 3.12–4.61) in Model IV; p for trend < 0.001) (**Table 3**). In the head-to-head comparison, the AUC of TyG-BMI for hypertension was 0.7087 and that of TyG 0.6250 (DeLong test Z = -12.28, p < 0.001; **Supplementary Table S3**; **Supplementary Figure S1**).

**Table 3:** Associations of TyG-BMI with hypertension in children and adolescents.

Model	Contrast	OR (95% CI)	p value
Model I	Per 1 SD	2.071 (1.989–2.157)	<0.001
	Q2 vs Q1	1.10 (0.91–1.34)	0.307
	Q3 vs Q1	1.88 (1.58–2.23)	<0.001
	Q4 vs Q1	5.33 (4.57–6.21)	<0.001
	p for trend		<0.001
Model II	Per 1 SD	2.121 (2.031–2.214)	<0.001
	Q2 vs Q1	1.15 (0.95–1.41)	0.150
	Q3 vs Q1	2.04 (1.69–2.45)	<0.001
	Q4 vs Q1	5.62 (4.74–6.66)	<0.001
	p for trend		<0.001
Model III	Per 1 SD	2.108 (1.985–2.238)	<0.001
	Q2 vs Q1	1.01 (0.83–1.23)	0.930
	Q3 vs Q1	1.69 (1.39–2.05)	<0.001
	Q4 vs Q1	3.74 (3.08–4.55)	<0.001
	p for trend		<0.001
Model IV	Per 1 SD	2.109 (1.985–2.241)	<0.001
	Q2 vs Q1	1.03 (0.84–1.26)	0.768
	Q3 vs Q1	1.73 (1.42–2.09)	<0.001
	Q4 vs Q1	3.79 (3.12–4.61)	<0.001
	p for trend		<0.001

OR, odds ratio; CI, confidence interval. Model I, unadjusted; Model II, adjusted for age, sex, smoking, alcohol use and milk tea consumption; Model III, Model II plus heart rate, low- and high-density lipoprotein cholesterol, total cholesterol, total protein, serum creatinine, uric acid and haemoglobin; Model IV, Model III plus workday and weekend sleep duration, workday and weekend sedentary time, moderate-to-vigorous physical activity, physical exercise, walking volume, snoring and family history of hypertension. Quartiles were defined on the corrected exposure distribution. Hypertension was defined using the Chinese reference values and the mean of the last two of three measurements.

### Non-linear associations and heart rate

Restricted cubic spline analysis (**Figure 2**) showed a monotonic positive association between TyG and hypertension that departed modestly from linearity (p for non-linearity 0.004) and no evidence of non-linearity for TyG-BMI (p for non-linearity 0.346); neither curve had an internal minimum. For heart rate, the spline fitted with the lowest AIC indicated a markedly non-linear association with hypertension (p for non-linearity < 0.001); the curve was essentially flat, with an OR close to 1.0, up to approximately 70 beats per minute and then increased steeply, reaching an OR of about 4.5 at 120 beats per minute (**Figure 2C**). No internal minimum was identified within the observed range, and in a grid search over 60–90 beats per minute, selecting the cut-point that minimised the AIC of a model containing the TyG × heart rate interaction, the AIC was lowest at the upper limit of the grid, so no interior cut-point was identified. Heart rate was therefore not dichotomised for the main regression models; the descriptive strata reported below were defined at 71 beats per minute, the 10th percentile of the observed distribution being 71.5 beats per minute. The subgroup estimates by sex and by heart-rate category, together with the quartile-specific estimates, are shown in **Figure 3** for TyG and **Figure 4** for TyG-BMI.

**Figure 2:**
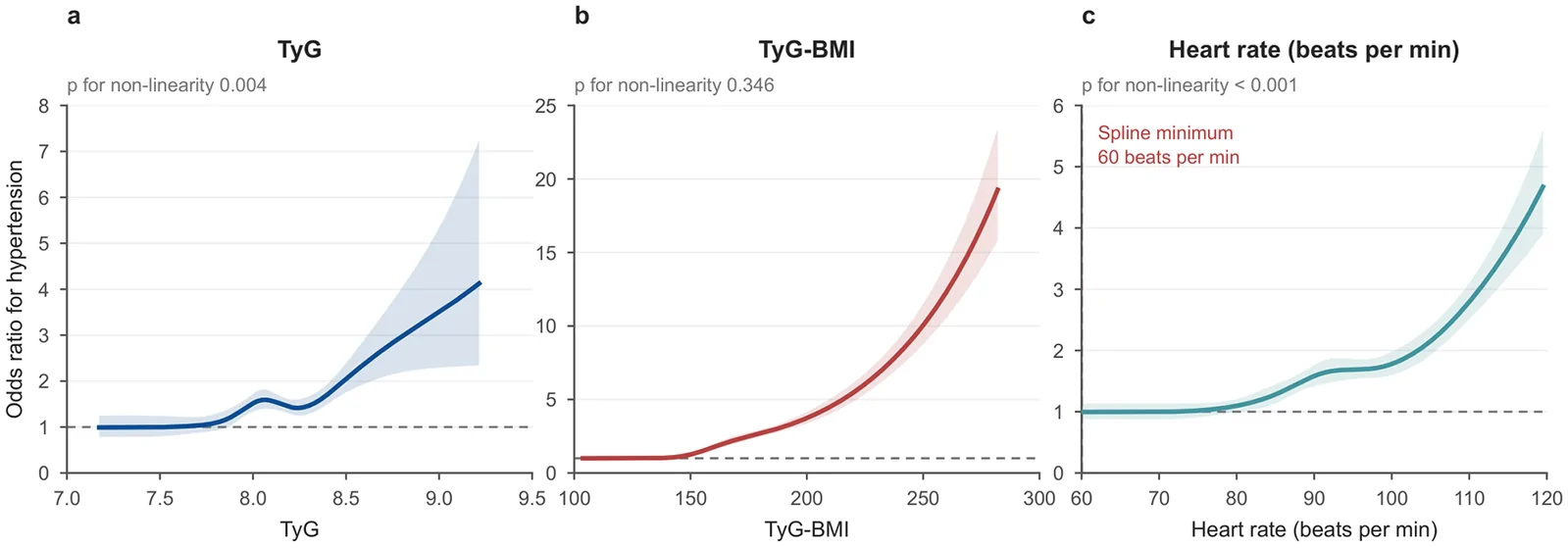
Restricted cubic spline analyses of the associations of TyG, TyG-BMI and heart rate with hypertension. Restricted cubic splines with the number of knots selected by the Akaike information criterion, adjusted for age, sex, smoking, drinking, milk tea, heart rate, lipids, total protein, serum creatinine, uric acid and hemoglobin. Shaded bands are 95% confidence intervals; the dashed horizontal line is the null (odds ratio 1). The reference value is the 10th percentile of the exposure. **(a)** TyG; **(b)** TyG-BMI; **(c)** heart rate.

**Figure 3:**
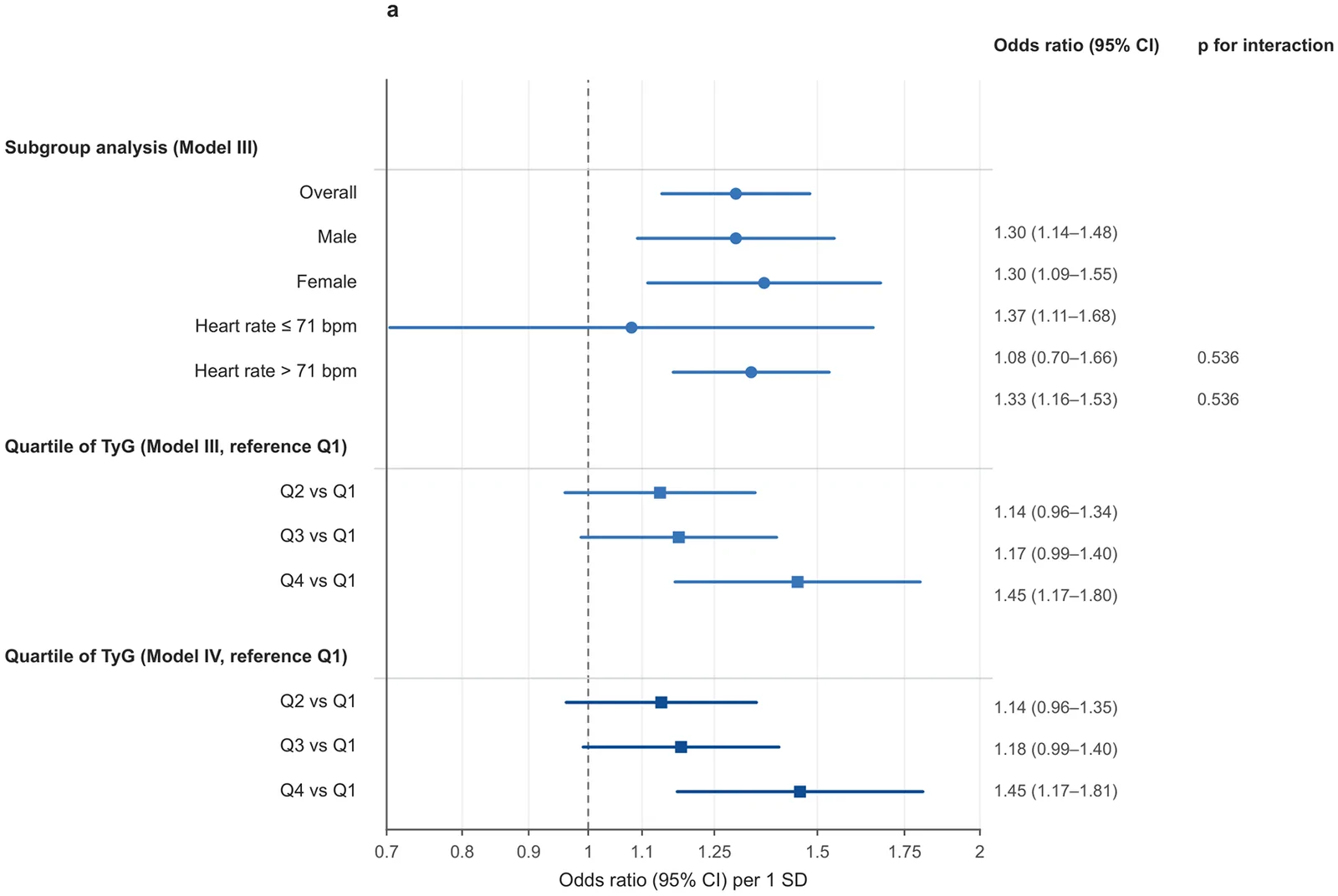
Subgroup and quartile analyses of the association between TyG and hypertension. OR, odds ratio; CI, confidence interval; bpm, beats per minute. Estimates are odds ratios per 1 SD of the exposure. Model III was adjusted for age, sex, smoking, drinking, milk tea, heart rate, lipids, total protein, serum creatinine, uric acid and hemoglobin; Model IV was additionally adjusted for sleep duration, sedentary time, physical activity, walking, snoring and family history of hypertension. Per-1-SD contrasts are shown as circles and quartile contrasts as squares. p for interaction is from the multiplicative interaction term between TyG and heart rate. Subgroup and interaction analyses are exploratory.

**Figure 4:**
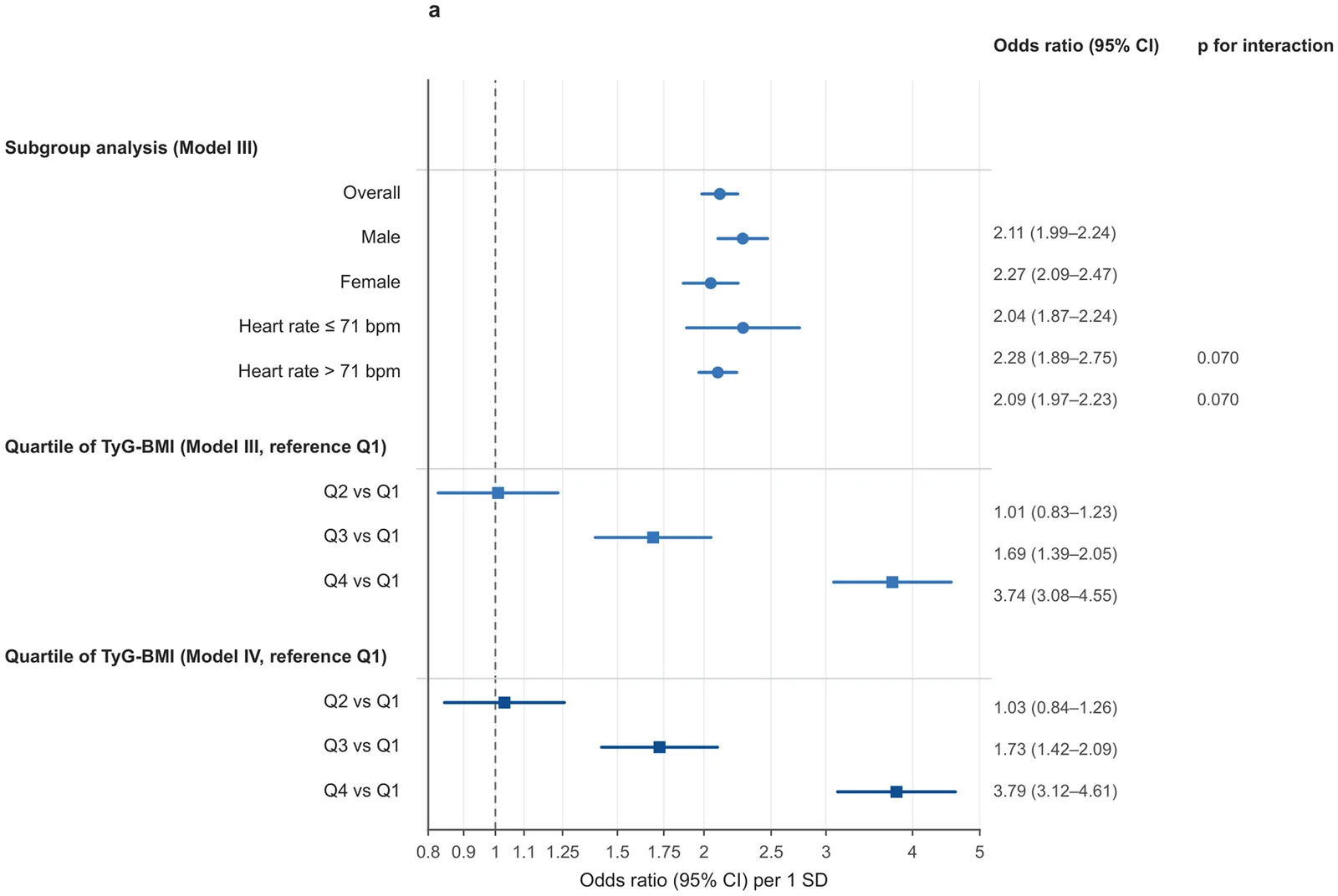
Subgroup and quartile analyses of the association between TyG-BMI and hypertension. Conventions, adjustment sets and abbreviations are as in **Figure 3**.

### Effect modification by central adiposity, uric acid and heart rate

Because the study has a cross-sectional design, the contributions of WHtR, uric acid and heart rate were evaluated as effect modifiers rather than as mediators. The stratified estimates were closely similar across strata and did not show a consistent direction: in Model III, the odds ratio per 1 SD of TyG was 1.27 above versus 1.39 below the median WHtR, 1.33 above versus 1.39 below the median uric acid and 1.33 above versus 1.08 at or below 71 beats per minute (the 10th percentile of the observed distribution), and the corresponding estimates for TyG-BMI showed a similar absence of a consistent pattern. The multiplicative and the additive scales are reported separately, because they answer different questions and are not interchangeable. On the multiplicative scale, the product term was not statistically significant for WHtR or for heart rate in either panel (p for interaction 0.156 and 0.536 in the TyG panel and 0.892 and 0.070 in the TyG-BMI panel), whereas the product term for uric acid was nominally significant in the TyG panel (p = 0.010) but not in the TyG-BMI panel (p = 0.423); after correction for the six interaction tests performed (Bonferroni-adjusted significance level 0.0083), no individual term remained significant. On the additive scale, RERI was positive with a 95% CI excluding zero for heart rate in both panels (TyG 0.148, 95% CI 0.044 to 0.253; TyG-BMI 0.444, 95% CI 0.314 to 0.575), for WHtR in the TyG panel (0.225, 95% CI 0.063 to 0.388) and for uric acid in both panels (TyG 0.206, 95% CI 0.100 to 0.311; TyG-BMI 0.100, 95% CI 0.024 to 0.176); the corresponding estimate for WHtR in the TyG-BMI panel was the smallest of the six (0.103, 95% CI 0.013 to 0.192). We therefore no longer state that there was no effect modification: the data provide no consistent evidence of interaction on the multiplicative scale, but several additive-scale estimates are positive, indicating departure from additivity. Because the study is cross-sectional, and because a RERI estimated from an odds-ratio model is constrained by the non-collapsibility of the odds ratio (21), these additive-scale findings are reported as exploratory and are not interpreted as evidence of biological interaction. The full set of multiplicative and additive estimates for all three modifiers and both exposures is given in **Table 4**, and the stratified estimates for waist-to-height ratio and uric acid are shown in **Figure 5**.

**Table 4:** Effect modification of the associations of TyG and TyG-BMI with hypertension by waist-to-height ratio, uric acid and heart rate.

Effect modifier	Exposure	Stratum	n	OR (95% CI) per 1 SD	p value	p for interaction	RERI (95% CI)
WHtR	TyG	Low	14784	1.39 (1.05–1.82)	0.019		
	TyG	High	11102	1.27 (1.08–1.50)	0.004		
		p for interaction				0.156	
		RERI (95% CI)					0.23 (0.06 to 0.39)
WHtR	TyG-BMI	Low	14784	1.93 (1.53–2.43)	<0.001		
	TyG-BMI	High	11102	2.00 (1.85–2.16)	<0.001		
		p for interaction				0.892	
		RERI (95% CI)					0.10 (0.01 to 0.19)
Uric acid	TyG	Low	13033	1.39 (1.08–1.79)	0.011		
	TyG	High	12853	1.33 (1.14–1.56)	<0.001		
		p for interaction				0.010	
		RERI (95% CI)					0.21 (0.10 to 0.31)
Uric acid	TyG-BMI	Low	13033	2.02 (1.79–2.28)	<0.001		
	TyG-BMI	High	12853	2.16 (2.01–2.32)	<0.001		
		p for interaction				0.423	
		RERI (95% CI)					0.10 (0.02 to 0.18)
Heart rate	TyG	above vs at or below the 10th percentile (stratified ORs in the text)	—	—	—		
		p for interaction				0.536	
		RERI (95% CI)					0.15 (0.04 to 0.25)
	TyG-BMI	above vs at or below the 10th percentile (stratified ORs in the text)	—	—	—		
	p for interaction				0.070	
	RERI (95% CI)					0.44 (0.31 to 0.57)

Participants were stratified at the median of waist-to-height ratio and of uric acid; for heart rate the strata were defined at 71 beats per minute (the 10th percentile of the observed distribution is 71.5 beats per minute), and only the interaction estimates are tabulated here, the stratified odds ratios being given in the text. ORs were estimated from Model III (see the note to **Table 2**). p for interaction was obtained from a likelihood ratio test comparing models with and without the product term between the standardised exposure and the standardised effect modifier; after correction for the six tests performed (Bonferroni level 0.0083), none of the multiplicative interactions remained significant. RERI, relative excess risk due to interaction, estimated on the additive scale with 95% confidence intervals obtained by the delta method. Because the study is cross-sectional, these are effect-modification analyses and not mediation analyses, and they are reported as exploratory.

**Figure 5:**
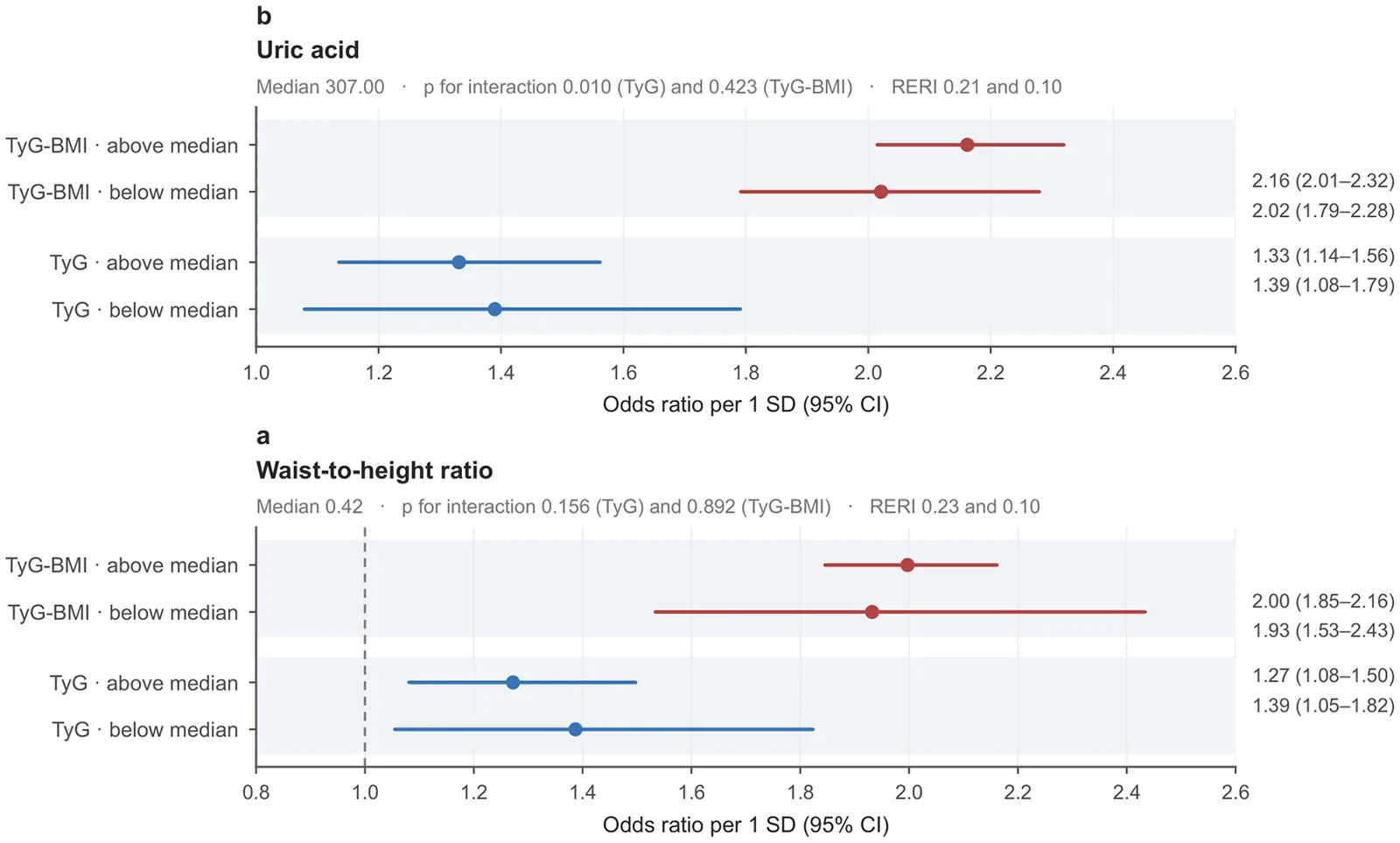
Effect modification of the associations of TyG and TyG-BMI with hypertension by waist-to-height ratio and uric acid. Strata were defined at the median of each effect modifier. OR, odds ratio; CI, confidence interval; RERI, relative excess risk due to interaction on the additive scale. p for interaction refers to the multiplicative interaction term. No multiplicative interaction term survived correction for the six tests performed, and the stratified estimates are therefore reported as exploratory. **(a)** Waist-to-height ratio; **(b)** uric acid.

In the descriptive strata, the OR per 1 SD of TyG was 1.33 (95% CI 1.16–1.53) in participants with a heart rate above 71 beats per minute and 1.08 (95% CI 0.70–1.66) in those at or below this value, and 2.09 (95% CI 1.97–2.23) versus 2.28 (95% CI 1.89–2.75) for TyG-BMI; the confidence interval for TyG in the lower stratum included unity, whereas that in the upper stratum did not. Subgroup analyses by sex and by heart-rate category showed positive associations in every subgroup, with overlapping confidence intervals (**Figures 3**, **4**; **Table 5**).

**Table 5:** Subgroup analyses of the associations of TyG and TyG-BMI with hypertension.

Subgroup	Exposure	n	OR (95% CI) per 1 SD	p value
Overall	TyG	25886	1.298 (1.139–1.480)	<0.001
	TyG-BMI	25886	2.108 (1.985–2.238)	<0.001
Male	TyG	13353	1.299 (1.091–1.546)	0.003
	TyG-BMI	13353	2.275 (2.094–2.471)	<0.001
Female	TyG	12533	1.365 (1.111–1.678)	0.003
	TyG-BMI	12533	2.045 (1.866–2.241)	<0.001
HR ≤ 71	TyG	2563	1.080 (0.704–1.656)	0.726
	TyG-BMI	2563	2.277 (1.887–2.748)	<0.001
HR > 71	TyG	23323	1.334 (1.162–1.532)	<0.001
	TyG-BMI	23323	2.094 (1.966–2.230)	<0.001

ORs were estimated from Model III (see the note to **Table 2**) within each subgroup. HR, heart rate; the heart-rate strata are descriptive and were defined at 71 beats per minute, the 10th percentile of the observed distribution being 71.5 beats per minute. This cut-point is not a threshold derived from the restricted cubic spline analysis: a grid search over 60–90 beats per minute found the AIC to be lowest at the upper limit of the grid, so no interior cut-point was identified and heart rate was not dichotomised in the main models.

### Sensitivity analyses

When the two alternative outcome definitions were used, the direction and statistical significance of the associations were unchanged (**Supplementary Table S1**). For the definition based on the Chinese reference and the mean of all three measurements (prevalence 8.30%), each 1-SD increment in TyG gave an OR of 1.208 (95% CI 1.070–1.365) and TyG-BMI an OR of 2.146 (95% CI 2.027–2.272) in Model IV. For the definition based on the European reference (prevalence 5.27%), the corresponding ORs were 1.388 (95% CI 1.198–1.609) and 2.347 (95% CI 2.193–2.512). Exclusion of participants with implausible hemoglobin values did not materially change any estimate. When the 2,026 participants outside the reference age range were re-included, the estimates were virtually identical to those of the primary analysis (Model IV per 1 SD 1.347 (95% CI 1.186–1.529) for TyG and 2.087 (95% CI 1.967–2.215) for TyG-BMI; **Supplementary Table S7**).

The analytic cluster-sensitivity assessment (**Supplementary Table S9**) showed that the association with TyG-BMI remained statistically significant across the entire grid examined, including the most extreme combination (mean cluster size 400 with an intra-cluster correlation of 0.05, corresponding to a design effect of 20.95), whereas the association with TyG remained statistically significant for every combination with a design effect up to 3.97 — which covers intra-cluster correlations of 0.01–0.03 with mean cluster sizes of up to 100 — and became non-significant only for the combinations with the larger clusters and the highest assumed correlation. Ignoring clustering therefore narrows the confidence interval for TyG by a factor of between 1.136 and 4.577 over the range examined, and for TyG-BMI by the same factor; the qualitative conclusions are unchanged, but the confidence intervals reported above should be interpreted as somewhat too narrow, particularly for the smaller TyG effect.

In the sequential attenuation analysis (**Supplementary Table S8**), the odds ratio per 1 SD of TyG decreased from 1.298 (95% CI 1.139–1.480) in the fully adjusted model to 1.204 (95% CI 1.053–1.378) after the addition of WHtR and to 1.158 (95% CI 1.011–1.326) after the further addition of BMI; the corresponding estimates for TyG-BMI were 2.108 (95% CI 1.985–2.238), 1.883 (95% CI 1.657–2.138) and 1.914 (95% CI 1.134–3.231). The associations therefore remained statistically significant after adjustment for central adiposity, but the attenuation indicates that part of the association is shared with adiposity. Because this analysis is descriptive, it does not partition the association into causal components.

### Feature selection and model performance

In the TyG panel, the pre-specified consensus rule selected 8 features: BMI, corpulence index, heart rate, LDL cholesterol, HDL cholesterol, uric acid, TyG, WHtR. In the TyG-BMI panel, 8 features were selected: corpulence index, heart rate, LDL cholesterol, HDL cholesterol, uric acid, BMI, gender, education (**Figure 6**; **Supplementary Table S5**). In both panels the strict intersection of the three algorithms was too small to serve as the predictor set (2 and 1 features, respectively), so the frequency-ranked fallback described in the Methods was applied. Neither exposure was selected by all three algorithms — TyG and TyG-BMI were each selected by Lasso and by Boruta but not by SVM-RFE — and the frequency-ranked rule handled this differently in the two panels. In the TyG panel exactly eight features reached a selection frequency of two or more, so the rule was unambiguous and TyG was included among the eight predictors of that model. In the TyG-BMI panel ten features reached that frequency while the rule retains eight, so two had to be dropped; the tie-break excluded TyG-BMI, and the TyG-BMI panel model therefore contains neither index. The two panels are thus not equivalent in this respect: TyG is one of the eight predictors of the TyG-panel model, whereas the TyG-BMI panel model is built from the remaining variables only, and the SHAP and LIME results presented below should be read accordingly.

**Figure 6:**
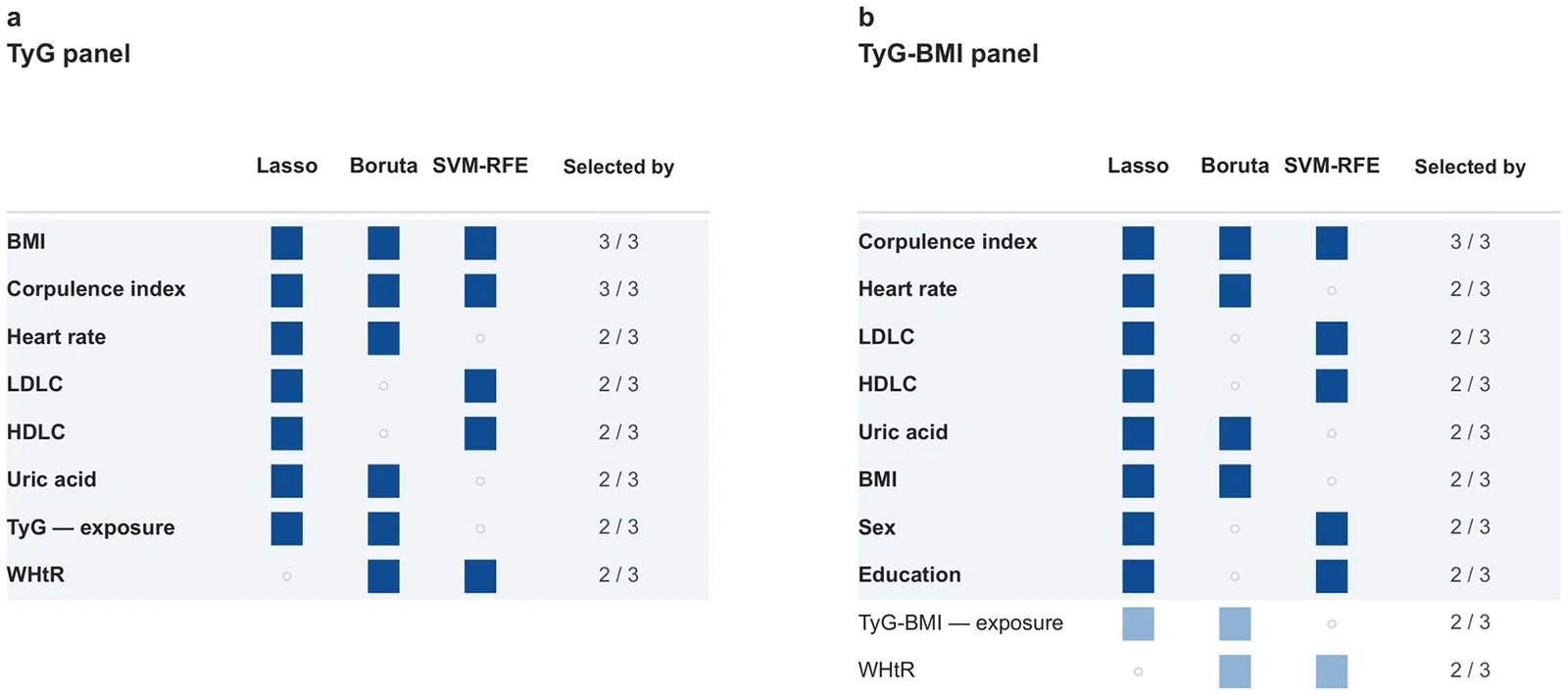
Feature selection within the training set: consensus across Lasso, Boruta and SVM-RFE. Filled squares indicate that the algorithm retained the feature within the training set. The final predictor set (upper block, shaded) comprises the eight features selected most frequently across the three algorithms; unshaded rows are further features selected by at least two of the three algorithms, shown for completeness. A pre-specified rule based on the strict intersection of all three algorithms could not be applied, because that intersection contained fewer than four features in both panels (two in the TyG panel and one in the TyG-BMI panel: BMI and corpulence index in the TyG panel, corpulence index in the TyG-BMI panel). Neither exposure was retained by all three algorithms. HDLC and LDLC, HDL and LDL cholesterol; HR, heart rate; UA, uric acid; SVM-RFE, support vector machine recursive feature elimination; WHtR, waist-to-height ratio. **(a)** TyG panel; **(b)** TyG-BMI panel.

In the held-out validation set, discrimination was moderate for most of the eleven algorithms, with AUCs between 0.55 and 0.75 (**Figure 7**; **Supplementary Table S6**); the linear support vector machine was the exception and performed close to chance (AUC 0.5550 in the TyG panel and 0.5809 in the TyG-BMI panel), which is expected when this algorithm is applied without kernel transformation to a strongly imbalanced dataset. In the TyG panel the best-performing model was XGBoost (AUC 0.7544, 95% CI 0.7302–0.7777); in the TyG-BMI panel the best model was XGBoost (AUC 0.7534, 95% CI 0.7298–0.7759). Mean AUCs during nested cross-validation within the training set were 0.7298 and 0.7342, respectively, indicating no substantial optimism. Calibration curves deviated only modestly from the ideal line (**Supplementary Figure S2**) and Brier scores ranged from 0.0575 to 0.0638. Decision curve analysis showed that most models provided a net benefit over the treat-all and treat-none strategies across threshold probabilities of approximately 5 to 25%, although the benefit was small in absolute terms (**Supplementary Figure S3**).

**Figure 7:**
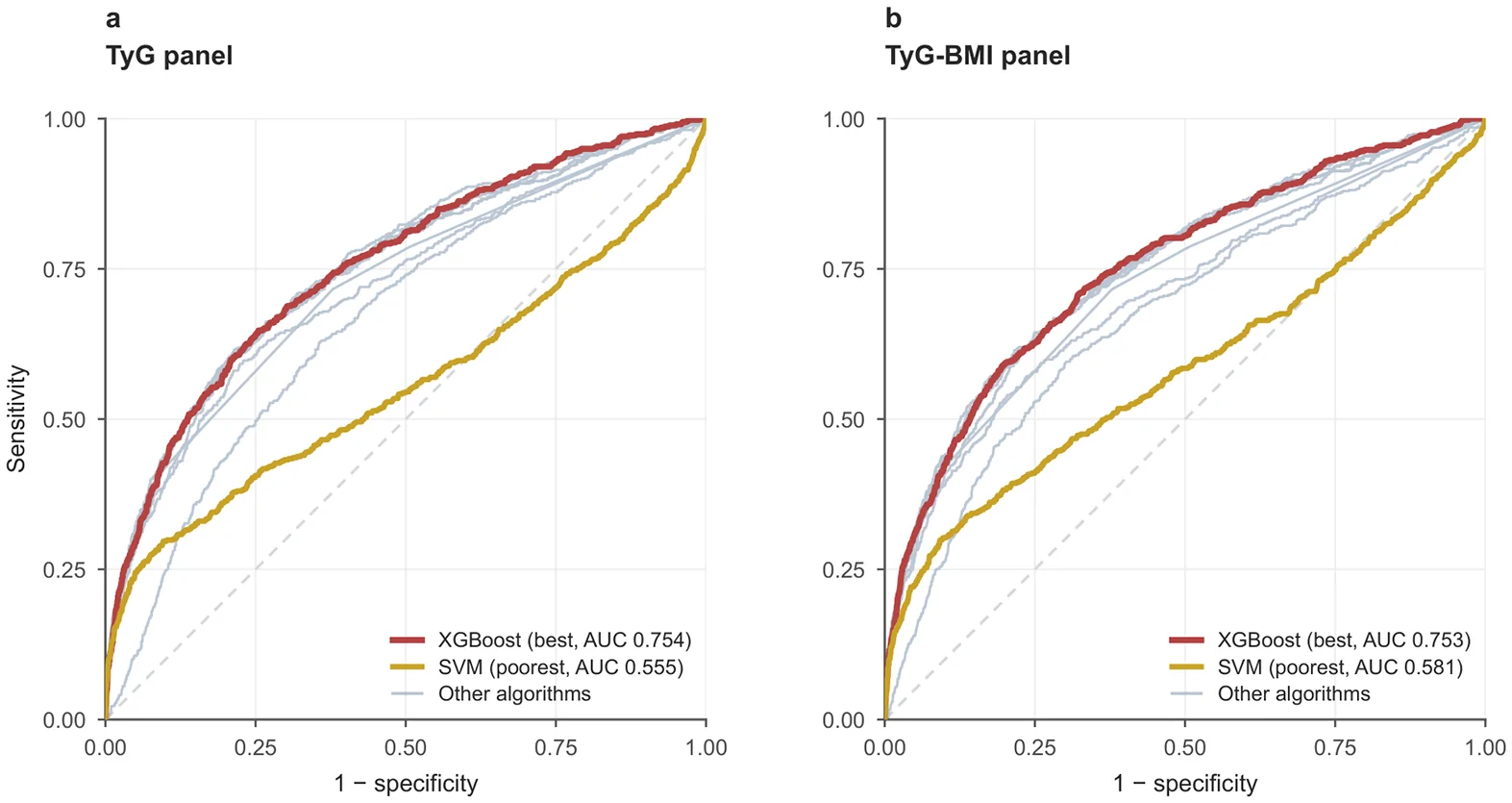
Discrimination of eleven machine-learning models in the held-out validation set. Receiver-operating characteristic curves in the held-out validation set (30% of the cohort). All eleven algorithms are shown; the best-performing model in each panel is highlighted in red and the poorest-performing model in gold. The dashed diagonal is the line of no discrimination. AUCs with 95% confidence intervals for every model are given in **Supplementary Table S6**. **(a)** TyG panel; **(b)** TyG-BMI panel.

Because the outcome is imbalanced, the classification metrics obtained at the default threshold of 0.50 are dominated by the majority class: sensitivity ranged from 0.000 to 0.058 across the eleven algorithms while specificity remained close to 1 (**Supplementary Table S6**). At the Youden-optimal threshold, which was selected in the same validation set in which these metrics were calculated, sensitivity rose to 0.297–0.716 and specificity fell to 0.622–0.908; the numeric threshold used for each model is reported in **Supplementary Table S6**. These threshold-dependent metrics are therefore reported as exploratory. Discrimination, as quantified by the AUC and the Brier score, is independent of the threshold, so the very low sensitivities obtained at 0.50 reflect the threshold and the low prevalence of the outcome rather than an absence of discriminative ability; they nevertheless mean that these models, as calibrated here, cannot be used for screening, and any clinical use would require an explicit choice of threshold based on the relative costs of false-negative and false-positive results.

To determine whether either index adds predictive value over an otherwise identical model, one common baseline was constructed from the intersection of the features selected in the two panels after removing the two indices themselves; the baseline therefore contains BMI (six predictors: BMI, corpulence index, heart rate, LDL cholesterol, HDL cholesterol and uric acid). The baseline and three augmented models — baseline + TyG, baseline + TyG-BMI, and baseline + both — were fitted on the same training set with the same training-set-fitted imputation and compared in the same held-out validation set with the DeLong test for correlated curves (**Supplementary Table S10**). The baseline reached an AUC of 0.7465; adding TyG raised it to 0.7493 (difference +0.0028, 95% CI + 0.0001 to +0.0055; DeLong p = 0.040), adding TyG-BMI raised it to 0.7486 (difference +0.0021, 95% CI -0.0001 to +0.0043; p = 0.056), and adding both raised it to 0.7495 (difference +0.0030, 95% CI + 0.0001 to +0.0059; p = 0.040). The increments are statistically detectable but very small — about 0.003 in AUC, that is, roughly three additional correctly ordered pairs in every 1,000 comparisons of a case with a non-case — and they do not change the classification performance of the models. We therefore describe the added value of both indices as marginal rather than absent, and we make no claim that either index should be used for prediction.

Because feature-selection frequencies were not unique at the retention threshold, we also examined how much the reported performance depends on the rule used to break ties. In the TyG panel exactly eight features reached a frequency of two or more, so the rule was unambiguous and TyG itself was retained. In the TyG-BMI panel ten features reached that frequency while the rule retains eight, so two had to be dropped; ties were broken by the order in which features first appeared in the concatenated output of the three algorithms, which excluded waist-to-height ratio and TyG-BMI. Enumerating all 36 ways of choosing eight features from that tied pool changed the validation AUC from 0.7383 to 0.7539, and the median AUC was 0.7507 for subsets that contained TyG-BMI and 0.7502 for those that did not; with the same XGBoost specification used in **Supplementary Table S6**, replacing education with TyG-BMI changed the AUC from 0.7534 to 0.7531, and adding TyG-BMI as a ninth predictor changed it to 0.7571 (**Supplementary Table S11**). The tie-break therefore has a negligible effect on model performance, and the inclusion or exclusion of TyG-BMI does not by itself explain the discrimination achieved.

### Model interpretation with SHAP and LIME

LIME explanations for individual participants highlighted the same variables that the global analysis identified as the most influential features (**Figure 8**). The distribution of SHAP values across the validation set is summarized in **Figure 9**, and dependence plots showed broadly monotonic increases in the SHAP values of the most influential features across their observed ranges, whereas the remaining variables made smaller and flatter contributions (**Figure 10**; **Supplementary Figure S4**). Global importance based on mean absolute SHAP values (**Figure 11**) ranked BMI, HR and CI as the most influential features in the TyG panel, with mean absolute SHAP values of 0.4341, 0.2892 and 0.2255, respectively.

**Figure 8:**
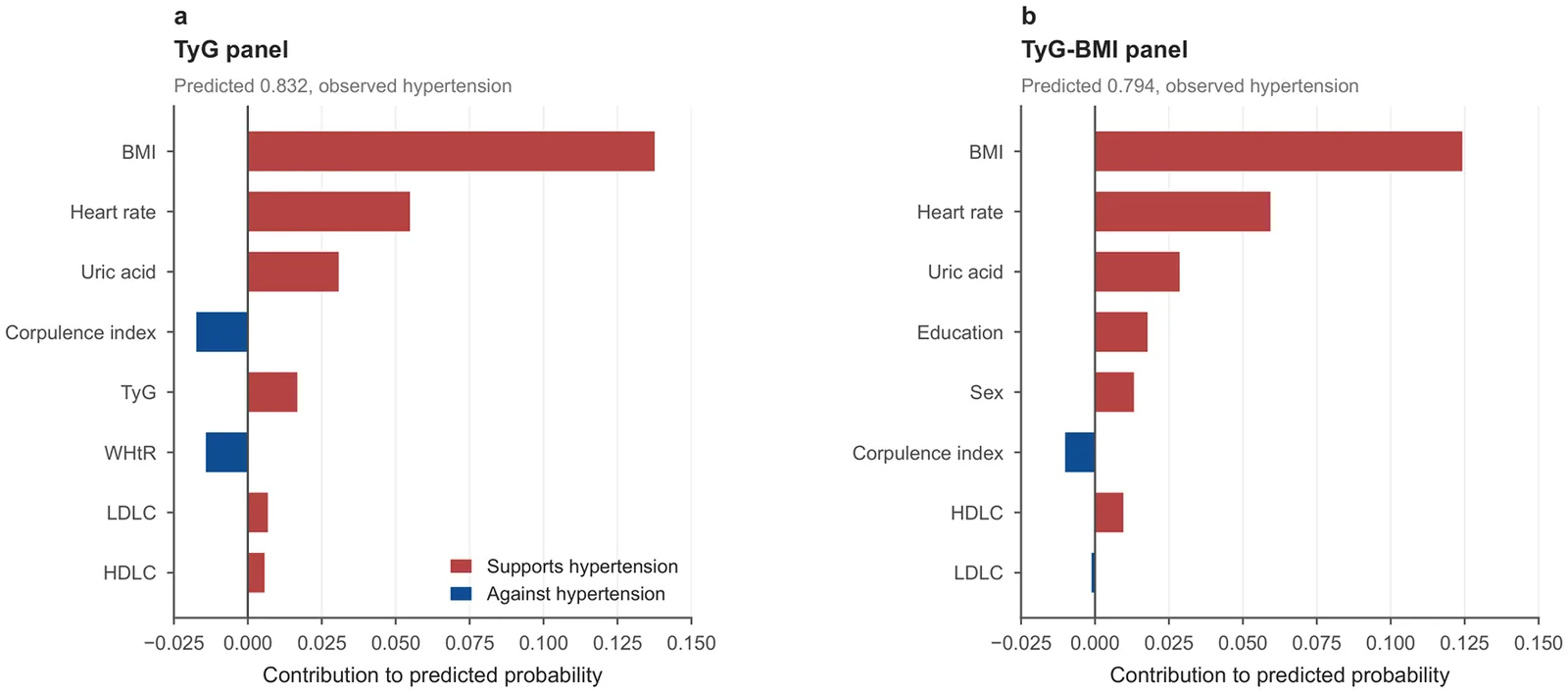
LIME explanations for individual predictions. LIME explanations for one individual participant in each panel, showing the eight features with the largest absolute contribution to the predicted probability. LIME discretizes continuous features into ranges; only the variable name is shown. LIME, local interpretable model-agnostic explanations. **(a)** TyG panel; **(b)** TyG-BMI panel.

**Figure 9:**
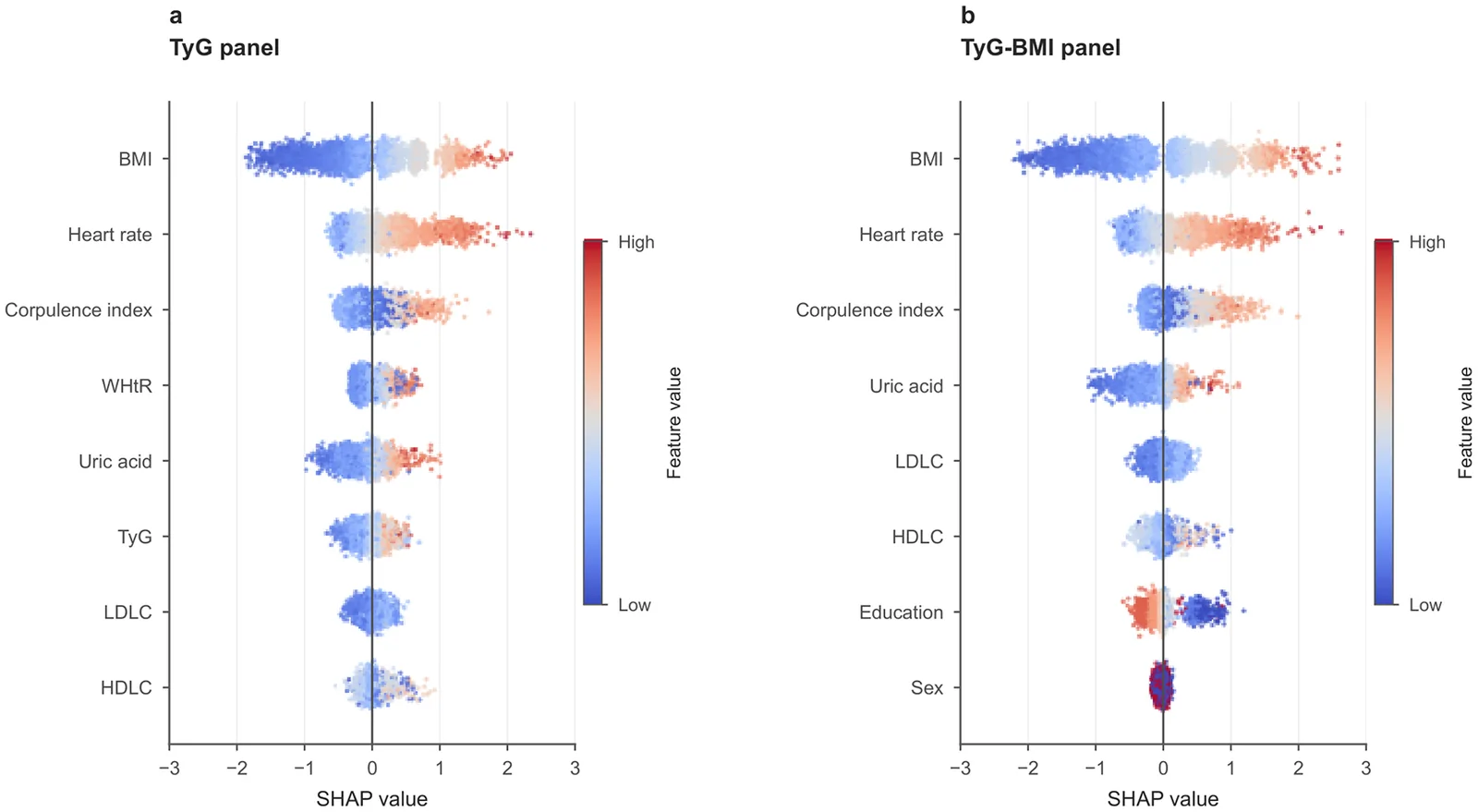
SHAP summary plots of the best-performing tree-based model in each panel. Each point is one participant in the validation set; the horizontal position is the SHAP value (the contribution of that feature to the predicted probability) and the color encodes the feature value. All eight predictor variables are shown. SHAP, Shapley additive explanations. **(a)** TyG panel; **(b)** TyG-BMI panel.

**Figure 10:**
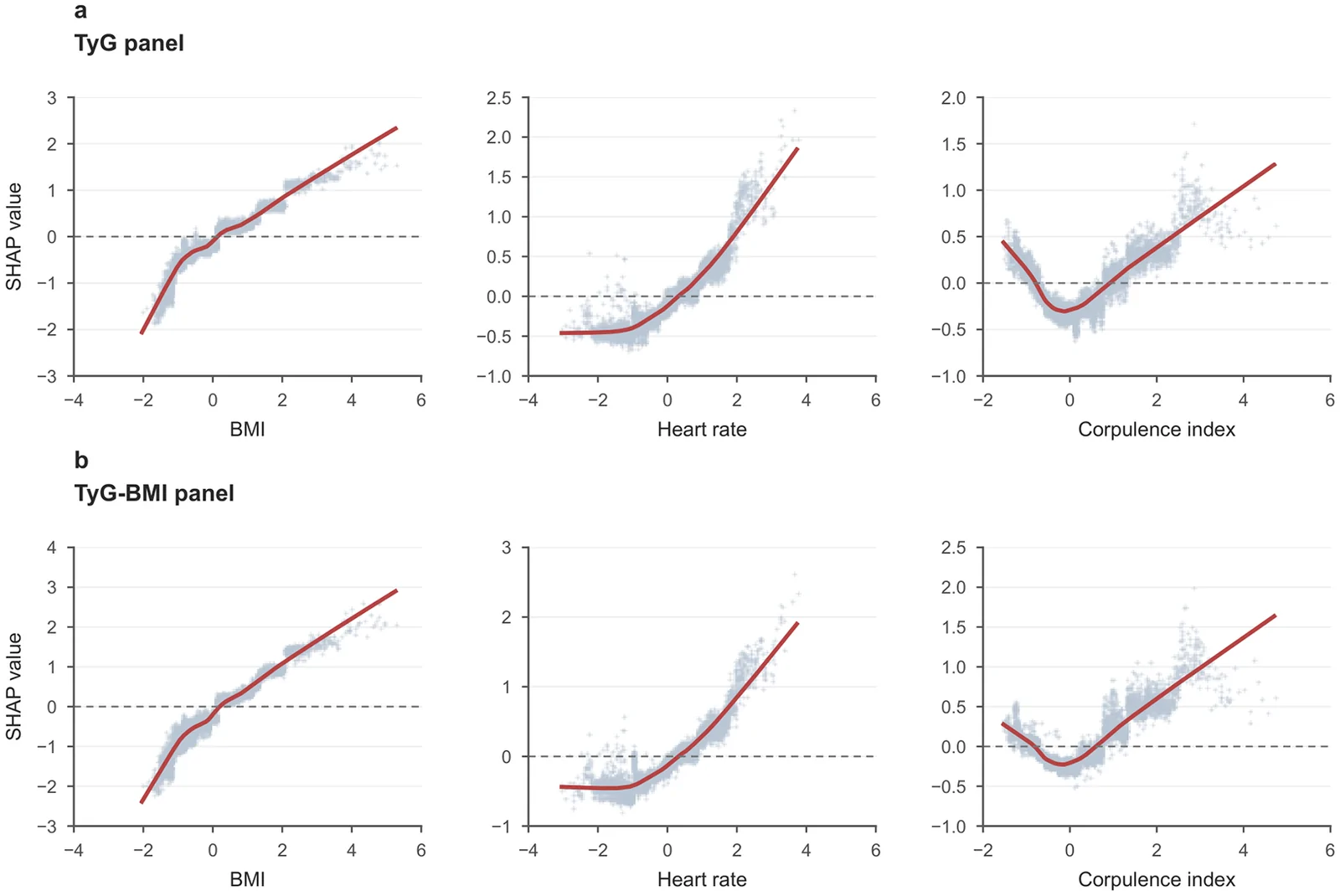
SHAP dependence plots for the three most important features in each panel. Points are individual participants; the red line is a locally weighted regression (LOWESS) trend. SHAP, Shapley additive explanations. **(a)** TyG panel (upper row); **(b)** TyG-BMI panel (lower row).

**Figure 11:**
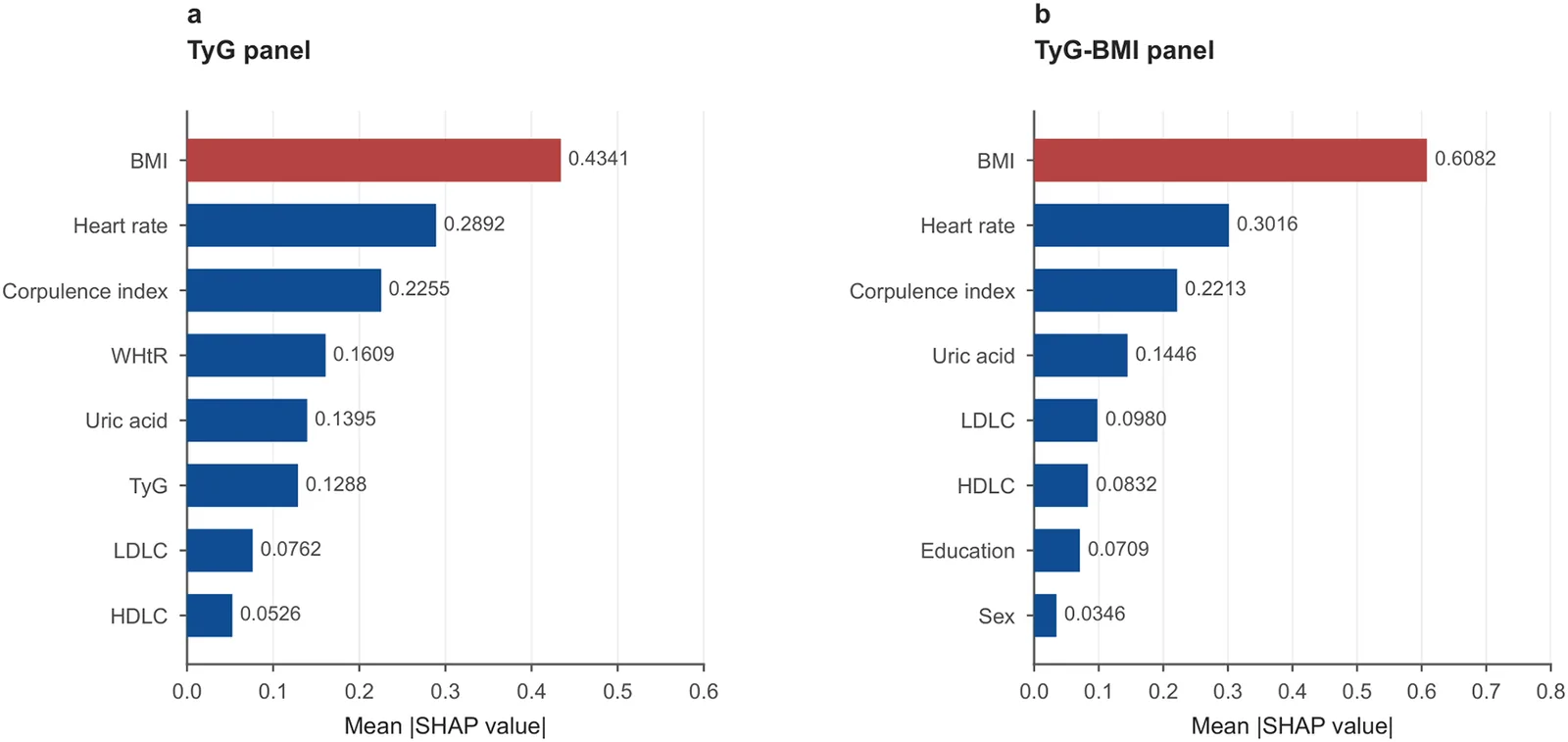
Global feature importance based on SHAP values. Global feature importance measured by the mean absolute SHAP value in the validation set. All eight predictor variables are shown. SHAP, Shapley additive explanations. **(a)** TyG panel; **(b)** TyG-BMI panel.

## Discussion

In this large cross-sectional survey of 25,886 Chinese children and adolescents aged 7–17 years, higher TyG and TyG-BMI were associated with a higher prevalence of hypertension, and the associations were robust to adjustment for demographic, lifestyle, biochemical and family-history covariates. The association was graded across quartiles and was stronger for TyG-BMI than for TyG. Stratified estimates were closely similar across strata and gave no consistent indication that the association differs according to central adiposity, uric acid or heart rate, and none of the formal multiplicative interaction tests was statistically significant, so these analyses are therefore exploratory. Machine-learning models built from routinely available variables achieved moderate discrimination, and SHAP analysis identified BMI, HR and CI as the most influential features. Because the design is cross-sectional, these findings describe associations and generate hypotheses; they cannot establish temporal order or causality.

The TyG index integrates triglycerides and fasting plasma glucose and thus provides a practical surrogate for insulin resistance. Insulin resistance may be particularly relevant in early life: because children have not yet accumulated the chronic structural and functional consequences of metabolic disturbance, the associated changes in endothelial function and sympathetic activity may be more reversible than in adults. In our data, both TyG and TyG-BMI were higher in participants with hypertension and the associations persisted after comprehensive adjustment. TyG-BMI incorporates BMI and therefore captures adiposity in addition to glucose–lipid dysregulation (28). As obesity is a central risk factor for childhood hypertension, and is already associated with adverse cardiovascular and renal measures in youth (29), the stronger association observed for TyG-BMI is plausible.

Heart rate deserves a specific comment. The spline showed that the odds of hypertension were close to unity up to approximately 70 beats per minute and then rose steeply. However, when the interaction was tested formally, the multiplicative interaction between heart rate and the exposure was not statistically significant (p = 0.536 for TyG and p = 0.070 for TyG-BMI), and the AIC was lowest at the upper limit of the grid of cut-points searched, so no interior cut-point was identified. Heart rate was therefore not dichotomized for the main models, and the stratified estimates are presented as descriptive only. The additive-scale RERI was positive (0.15, 95% CI 0.04 to 0.25 for TyG), which is the pattern that would be expected if heart rate and insulin resistance contributed to blood pressure through partly shared pathways, but a cross-sectional design cannot distinguish this from confounding. Mechanistically, insulin resistance may increase sympathetic outflow, while sympathetic activation can in turn aggravate insulin resistance, producing a self-reinforcing cycle that promotes vasoconstriction and sodium retention (30–33); our data cannot establish the direction of this relationship.

We deliberately did not perform mediation analysis. In a cross-sectional study, WHtR and uric acid are measured at the same time as the exposure and the outcome, so the temporal ordering required for mediation cannot be established (21); mediation proportions estimated under these conditions are liable to bias and should not be interpreted as evidence of a mechanistic pathway. We therefore recast the corresponding analyses as effect-modification and attenuation analyses, and we report them as exploratory. The stratified estimates did not point in a consistent direction and give no support to the hypothesis that central adiposity amplifies the association: the odds ratio per 1 SD of TyG was, if anything, slightly lower in participants above the median WHtR than below it, and the same was true for uric acid in the TyG panel, whereas the opposite pattern was seen for uric acid with TyG-BMI and for heart rate with TyG. We therefore do not interpret these strata as evidence of effect modification (34, 35). We do, however, state the two scales separately rather than concluding that no interaction is present: on the multiplicative scale no product term survived correction for the six tests performed, whereas on the additive scale all six relative excess risk estimates were positive with confidence intervals excluding zero, although the estimate for waist-to-height ratio in the TyG-BMI panel was small (0.10, 95% CI 0.01 to 0.19). Because the odds ratio is non-collapsible, an additive-scale departure from additivity can coexist with a null multiplicative product term, so the two scales answer different questions and neither is intrinsically more correct; on cross-sectional data neither can be read as biological interaction. The substantial association between TyG-BMI and hypertension in particular indicates that the combination of adiposity and glucose-lipid dysregulation identifies a high-risk group. The uric acid findings should be read as the weakest of the three: the multiplicative interaction in the TyG panel was nominally significant (p = 0.010) but did not survive the Bonferroni-corrected significance level of 0.0083, and the additive-scale excess risk was small; hyperuricemia may be a marker of metabolic dysfunction rather than a driver (36, 37).

Our findings are consistent with the small number of comparable pediatric studies, while differing in magnitude. In the 2017 China National Nutrition and Health Surveillance analysis of 12,087 children and adolescents, TyG was associated with isolated systolic, isolated diastolic and systolic–diastolic hypertension, with larger effect estimates than those reported here (16). Several differences plausibly explain the discrepancy. That analysis defined hypertension from a single set of measurements rather than from the mean of repeated measurements on non-consecutive days, which tends to raise the apparent prevalence and to inflate associations relative to our more conservative definition; it adjusted for a smaller covariate set, so that part of the association we attribute to adiposity and lifestyle factors may have remained in the exposure term; and it did not report effect-modification analyses, whereas our estimates are presented separately for strata of WHtR, a correlate of both TyG and blood pressure. Where the two studies agree is in the direction of the association and in the graded, dose-dependent pattern. Our results are also consistent in direction with adult studies of the TyG index and hypertension (28), although effect sizes are not directly comparable because of the different outcome definitions and age ranges.

The machine-learning analyses should be interpreted in the light of their moderate discriminatory performance. A 2025 population-based cohort study in Hong Kong reported AUCs of 0.91–0.92 for the prediction of blood pressure status one and three years later using 36 easily assessed predictors (38). That figure is substantially higher than ours, but the two studies answer different questions. That study predicted future blood pressure status in a longitudinal screening program, with the outcome measured at a later visit; our models estimate the probability that a child has hypertension at the time of assessment, from predictors measured simultaneously. In a cross-sectional setting, the achievable discrimination is intrinsically limited, and the best models reported here achieved an AUC of about 0.75 in each panel, which is in keeping with the modest discrimination typically achieved by cross-sectional pediatric hypertension models. We therefore make no claim of clinical utility: this level of discrimination indicates that the models can rank individuals but is not sufficient for them to serve as stand-alone screening tools, and they should be regarded as hypothesis-generating contributions to risk stratification. Our results therefore should not be read as a claim that machine learning is inferior to simple regression for this task; rather, they indicate that the information carried by routinely available anthropometric, biochemical and lifestyle variables is not sufficient for accurate case identification in a single-cross-section design.

Several design choices increase confidence in the estimates we do report. Imputation and feature selection were performed within the training set only, so that information from the validation set could not influence the predictor set; hyperparameters were tuned by nested cross-validation; and the models were evaluated once in a held-out validation set. The close agreement between the nested cross-validation and the validation-set AUCs indicates that the reported performance is not materially optimistic. The frequency-ranked consensus across Lasso, Boruta and SVM-RFE further reduces the dependence of the predictor set on the properties of any single algorithm, since each has distinct limitations: Lasso may select arbitrarily among correlated predictors, Boruta captures non-linear associations, and SVM-RFE is sensitive to the decision boundary (24–26, 39).

The interpretability analyses identified BMI, HR and CI as the leading contributors to the predictions, consistent with the regression and effect-modification results and with a neuro-metabolic–adiposity framework (40–42). It should be emphasized that SHAP and LIME describe how a given model partitions information, not the biological importance of a variable, and they cannot substitute for external validation.

### Limitations

Several limitations should be acknowledged. First, the cross-sectional design precludes any conclusion about temporal order or causality; all estimates describe associations, and prospective cohort studies are needed to determine whether changes in TyG precede the development of hypertension. Second, the de-identified dataset contained neither school nor city identifiers, so the clustering of participants could not be modelled directly. We therefore quantified the consequences analytically: across a grid of mean cluster sizes (30–400) and intra-cluster correlations (0.01–0.05), the association with TyG-BMI remained statistically significant in every scenario, whereas the association with TyG remained significant in 12 of the 20 scenarios, up to a design effect of 3.97; solving for the design effect at which its p value would reach 0.05 gives DE* = 4.12, so the TyG association is robust to clustering unless the product of the average cluster size and the intra-cluster correlation is unusually large. The confidence intervals reported here should therefore be regarded as somewhat too narrow, particularly for TyG, although the point estimates themselves are unaffected. Third, although the sample includes 18 prefecture-level cities, all participants were recruited in Henan Province, and validation in a nationally representative multicenter sample is required. Fourth, detailed dietary exposures such as sodium and fructose intake were not available, which may have led to residual confounding of the diet-related component of the association. Fifth, the effect-modification analyses were exploratory: no multiplicative product term survived correction for the six tests performed, whereas several additive-scale estimates were positive. Because the multiplicative and additive scales can diverge through the non-collapsibility of the odds ratio, we do not conclude that no interaction is present; on cross-sectional data, however, neither scale can be read as biological interaction. Sixth, the prediction models achieved only moderate discrimination and have not been externally validated, and at the default probability threshold their sensitivity was very low; they should not be used for clinical decision-making at this stage. The classification metrics reported at the Youden-optimal threshold are exploratory, because that threshold was selected in the same validation set in which the metrics were then calculated; the threshold-independent measures (AUC, Brier score and the calibration curves) are therefore the results on which the performance of these models should be judged. The two panels also differ in one respect that should be borne in mind when they are compared: the TyG-panel model contains TyG among its eight predictors, whereas the TyG-BMI panel model contains neither index. Seventh, because the reference values used to define hypertension are tabulated only for children and adolescents aged 7–17 years, and because the thresholds applied outside that range could not be verified from the de-identified dataset, the primary analysis was restricted to 7–17 years; the findings therefore do not extend to younger children or to adults, and the 2,026 participants outside this range were examined only in a sensitivity analysis (**Supplementary Table S7**). Eighth, the de-identified dataset available for the present analysis contains the derived hypertension indicator rather than the individual blood-pressure readings, so the averaging rule and the reference values could not be rederived or audited independently, and the measurement schedule could not be verified against source records; the outcome definition rests on the derivation documented in that dataset.

## Conclusion

In this large cross-sectional study of Chinese children and adolescents aged 7–17 years, higher TyG and TyG-BMI were associated with a higher prevalence of hypertension, and the association was stronger for TyG-BMI. The stratified estimates were closely similar across strata, and no multiplicative interaction term survived correction for multiple testing; several additive-scale estimates were nevertheless positive, so effect modification cannot be excluded and these analyses remain exploratory. Neither index added more than a marginal increment in discrimination when it was added to the same baseline model that already contained BMI (about 0.003 in AUC). Machine-learning models built from routinely available variables showed moderate discrimination and should be regarded as hypothesis-generating contributions to risk stratification rather than as validated clinical tools. Longitudinal studies with cluster-level information and detailed dietary assessment are required to clarify whether these associations are causal and to establish their clinical utility.

## Data Availability

The original contributions presented in the study are included in the article/**Supplementary Material**. Further inquiries can be directed to the corresponding authors.

## Supplementary material

Supplementary Figure 1 Head-to-head comparison of TyG and TyG-BMI for hypertension in children and adolescents. Panel a, discrimination of single biomarkers for hypertension; panel b, odds ratios per 1 SD of TyG and TyG-BMI across progressively adjusted models. Values in parentheses are 95% confidence intervals. The DeLong test for two correlated curves gave Z = −12.28 (p < 0.001) for TyG versus TyG-BMI. Model I was unadjusted; later models were adjusted progressively. AUC, area under the receiver-operating characteristic curve; ABSI, a body shape index; Hb, hemoglobin; WHtR, waist-to-height ratio.

Supplementary Figure 2 Calibration of eleven machine-learning models in the validation set. Calibration curves in the held-out validation set, with predicted probabilities grouped into ten quantile bins. The dashed diagonal represents perfect calibration. The best-performing model in each panel is highlighted in red and the poorest-performing model in gold.

Supplementary Figure 3 Decision curve analysis of eleven machine-learning models. Decision curve analysis in the held-out validation set. The horizontal gray line is the strategy of treating no participant and the dashed gray curve the strategy of treating all participants. The best-performing model in each panel is highlighted in red and the poorest-performing model in gold.

Supplementary Figure 4 SHAP dependence plots for the most important continuous features. Points are individual participants; the red line is a locally weighted regression (LOWESS) trend. SHAP, Shapley additive explanations.
