# Supplementary material for: Association between the triacylglycerol–glucose index, TyG-BMI and hypertension in children and adolescents: a cross-sectional study with machine-learning-based risk stratification
Source: Front Endocrinol (Lausanne). 2026 Oct 16;17:1964709. doi: 10.3389/fendo.2026.1964709 (PMC13719414; doi:10.3389/fendo.2026.1964709)
Supplement: Supplementary file 1 [file Supplementaryfile1.docx]

**Supplementary Tables**

**Table S1. Sensitivity analyses using three definitions of the primary outcome.**

| **Outcome definition** | **Exposure** | **Cases** | **Prevalence** | **OR (95% CI) per 1 SD** | **OR (95% CI), Q4 vs Q1** |
| --- | --- | --- | --- | --- | --- |
| Primary: Chinese reference, mean of the last two of three measurements | TyG | 1798 | 6.95% | 1.306 (1.145–1.489) | 1.45 (1.17–1.81) |
|  | TyG-BMI | 1798 | 6.95% | 2.109 (1.985–2.241) | 3.79 (3.12–4.61) |
| Sensitivity 1: Chinese reference, mean of all three measurements | TyG | 2148 | 8.30% | 1.208 (1.070–1.365) | 1.30 (1.06–1.59) |
|  | TyG-BMI | 2148 | 8.30% | 2.146 (2.027–2.272) | 4.18 (3.47–5.02) |
| Sensitivity 2: European reference, mean of all three measurements | TyG | 1364 | 5.27% | 1.388 (1.198–1.609) | 1.50 (1.17–1.92) |
|  | TyG-BMI | 1364 | 5.27% | 2.347 (2.193–2.512) | 5.77 (4.60–7.23) |

All estimates are from Model IV (see the note to Table 2). Blood pressure was measured three times on non-consecutive days and the mean of the last two measurements was used; the Chinese reference values are those of Huo et al. [15]. Because these reference values are tabulated only for ages 7–17 years, the analysis set for this table is restricted to that range (n = 25,886) and the 2,026 participants (7.26%) outside it were examined separately. In a sensitivity analysis that re-included those participants (total n = 27,912), the Model IV odds ratios per 1 SD were (1.19–1.53) for TyG and (1.97–2.21) for TyG-BMI, essentially unchanged (Table S7).

**Table S2. Restricted cubic spline analysis: model selection and tests for non-linearity.**

| **Exposure** | **Knots (AIC-optimal)** | **AIC** | **p for non-linearity** | **Nadir of the fitted curve** | **Reference (10th percentile)** |
| --- | --- | --- | --- | --- | --- |
| TyG | 7 | 12049.1 | 0.004 | 7.17 | 7.54 |
| TyG-BMI | 6 | 11472.8 | 0.346 | 103.66 | 118.45 |
| Heart rate | 6 | 12055.8 | <0.001 | 60.00 | 71.50 |

Cubic splines with three to seven knots were compared by the Akaike information criterion (AIC); the model with the lowest AIC was retained. Non-linearity was tested by a likelihood ratio test comparing the spline model with a model containing only a linear term. Models were adjusted as in Model III (see the note to Table 2). For heart rate the 10th percentile, which is the reference value of the spline, is 71.5 beats per minute; the descriptive heart-rate strata used in Tables 4 and 5 are defined at 71 beats per minute.

**Table S3. Head-to-head comparison of TyG, TyG-BMI and other biomarkers of hypertension.**

| **Biomarker** | **AUC** | **95% CI** |
| --- | --- | --- |
| TyG-BMI | 0.7087 | (0.6953–0.7219) |
| TyG | 0.6250 | (0.6104–0.6384) |
| BMI | 0.7039 | (0.6909–0.7179) |
| Waist-to-height ratio | 0.6947 | (0.6805–0.7084) |
| Uric acid | 0.6383 | (0.6249–0.6529) |
| Heart rate | 0.5557 | (0.5404–0.5693) |
| A body shape index | 0.5960 | (0.5829–0.6094) |
| Corpulence index | 0.3481 | (0.3339–0.3621) |
| Age | 0.5622 | (0.5486–0.5765) |
| Haemoglobin | 0.6200 | (0.6064–0.6337) |
| DeLong test: TyG vs TyG-BMI | Z = -12.28 | p < 0.001 |

AUCs are based on the raw biomarker values without covariate adjustment. Confidence intervals were obtained from 500 bootstrap resamples. The DeLong test accounts for the correlation between the two curves.

**Table S4. Tests for effect modification of the associations of TyG and TyG-BMI with hypertension by waist-to-height ratio, uric acid and heart rate.**

| **Effect modifier** | **Exposure** | **OR (95% CI) per 1 SD, lower stratum** | **OR (95% CI) per 1 SD, higher stratum** | **p for interaction** | **RERI (95% CI)** |
| --- | --- | --- | --- | --- | --- |
| Waist-to-height ratio | TyG | 1.387 (1.055–1.823) | 1.272 (1.081–1.497) | 0.156 | 0.23 (0.06 to 0.39) |
|  | TyG-BMI | 1.932 (1.534–2.434) | 1.997 (1.845–2.161) | 0.892 | 0.10 (0.01 to 0.19) |
| Uric acid | TyG | 1.390 (1.079–1.791) | 1.331 (1.135–1.562) | 0.010 | 0.21 (0.10 to 0.31) |
|  | TyG-BMI | 2.021 (1.792–2.279) | 2.162 (2.015–2.320) | 0.423 | 0.10 (0.02 to 0.18) |
| Heart rate | TyG | 1.080 (0.704–1.656) | 1.334 (1.162–1.532) | 0.536 | 0.15 (0.04 to 0.25) |
|  | TyG-BMI | 2.277 (1.887–2.748) | 2.094 (1.966–2.230) | 0.070 | 0.44 (0.31 to 0.57) |

Strata were defined at the median for waist-to-height ratio and uric acid and at 71 beats per minute for heart rate (the 10th percentile of the observed distribution is 71.5 beats per minute); the heart-rate strata are descriptive only. ORs were estimated from Model III (see the note to Table 2). p for interaction is from a likelihood ratio test comparing models with and without the product term between the standardised exposure and the standardised effect modifier; after correction for the six tests performed (Bonferroni level 0.0083), none of the multiplicative interactions remained significant. RERI, relative excess risk due to interaction, estimated on the additive scale, with 95% confidence intervals obtained by the delta method. Because the study is cross-sectional, these analyses are exploratory and are not mediation analyses.

**Table S5. Features selected by Lasso, Boruta and SVM-RFE within the training set.**

| **Panel** | **Feature** | **Lasso** | **Boruta** | **SVM-RFE** | **In final model** |
| --- | --- | --- | --- | --- | --- |
| TyG | ABSI |  |  | Yes |  |
|  | BMI | Yes | Yes | Yes | Yes |
|  | CI | Yes | Yes | Yes | Yes |
|  | HDLC | Yes |  | Yes | Yes |
|  | HR | Yes | Yes |  | Yes |
|  | Hem | Yes |  |  |  |
|  | LDLC | Yes |  | Yes | Yes |
|  | TC |  |  | Yes |  |
|  | TyG | Yes | Yes |  | Yes |
|  | UA | Yes | Yes |  | Yes |
|  | WHtR |  | Yes | Yes | Yes |
|  | alcohol | Yes |  |  |  |
|  | education | Yes |  |  |  |
|  | f_grandparents_hypertension |  |  | Yes |  |
|  | gender | Yes |  |  |  |
|  | mother_hypertension |  |  | Yes |  |
|  | weekenday_sedentary_d_h | Yes |  |  |  |
|  | workday_sedentary_d_h | Yes |  |  |  |
|  | workdaynightsleep_h |  |  | Yes |  |
|  | zongdanbai | Yes |  |  |  |
| TyG-BMI | BMI | Yes | Yes |  | Yes |
|  | CI | Yes | Yes | Yes | Yes |
|  | HDLC | Yes |  | Yes | Yes |
|  | HR | Yes | Yes |  | Yes |
|  | Hem | Yes |  |  |  |
|  | LDLC | Yes |  | Yes | Yes |
|  | TC |  |  | Yes |  |
|  | TyG_BMI | Yes | Yes |  |  |
|  | UA | Yes | Yes |  | Yes |
|  | WHtR |  | Yes | Yes |  |
|  | age |  | Yes |  |  |
|  | alcohol | Yes |  |  |  |
|  | education | Yes |  | Yes | Yes |
|  | f_grandparents_hypertension |  |  | Yes |  |
|  | gender | Yes |  | Yes | Yes |
|  | m_grandparents_hypertension |  |  | Yes |  |
|  | mother_hypertension |  |  | Yes |  |
|  | weekenday_sedentary_d_h | Yes |  |  |  |
|  | workday_sedentary_d_h | Yes |  |  |  |
|  | zongdanbai | Yes |  |  |  |

Feature selection was performed exclusively within the training set (70% of participants) using five-fold cross-validation. The final predictor set for each panel (rightmost column) was defined by a pre-specified two-step rule: features selected by all three algorithms were retained, and because this strict intersection contained fewer than four features in both panels, the rule fell back to the eight features selected most frequently across the three algorithms. Neither exposure was selected by all three algorithms. In the TyG panel exactly eight features reached a selection frequency of two or more, so the rule was unambiguous and TyG was retained and is one of the eight predictors of that model. In the TyG-BMI panel ten features reached that frequency while the rule retains eight, so two had to be dropped; the tie-break excluded TyG-BMI, and that panel therefore contains neither index.

**Table S6. Performance of the eleven machine-learning models in the held-out validation set.**

| **Panel** | **Model** | **AUC** | **95% CI** | **Nested CV AUC** | **Accuracy** | **Threshold @0.50 / @Youden** | **Sensitivity @0.50 / @Youden** | **Specificity @0.50 / @Youden** | **PPV** | **NPV** | **F1** | **Brier score** |
| --- | --- | --- | --- | --- | --- | --- | --- | --- | --- | --- | --- | --- |
| TyG panel | Logistic | 0.7495 | (0.7248–0.7730) | 0.7332 | 0.933 | 0.50 / 0.0766 | 0.052 / 0.614 | 0.999 / 0.786 | 0.737 | 0.934 | 0.097 | 0.0575 |
|  | DecisionTree | 0.7231 | (0.6990–0.7489) | 0.7018 | 0.931 | 0.50 / 0.0752 | 0.043 / 0.716 | 0.998 / 0.622 | 0.561 | 0.933 | 0.079 | 0.0586 |
|  | Lasso | 0.7495 | (0.7248–0.7729) | 0.7332 | 0.933 | 0.50 / 0.0766 | 0.052 / 0.616 | 0.999 / 0.786 | 0.737 | 0.934 | 0.097 | 0.0575 |
|  | Ridge | 0.7522 | (0.7286–0.7758) | 0.7338 | 0.933 | 0.50 / 0.0641 | 0.052 / 0.662 | 0.999 / 0.725 | 0.778 | 0.934 | 0.097 | 0.0575 |
|  | ENet | 0.7495 | (0.7248–0.7730) | 0.7332 | 0.933 | 0.50 / 0.0762 | 0.052 / 0.616 | 0.999 / 0.784 | 0.737 | 0.934 | 0.097 | 0.0575 |
|  | KNN | 0.7194 | (0.6937–0.7436) | 0.6902 | 0.931 | 0.50 / 0.0916 | 0.017 / 0.592 | 0.999 / 0.776 | 0.692 | 0.932 | 0.033 | 0.0594 |
|  | RandomForest | 0.7542 | (0.7305–0.7786) | 0.7297 | 0.932 | 0.50 / 0.0788 | 0.032 / 0.594 | 1.000 / 0.801 | 0.850 | 0.933 | 0.061 | 0.0577 |
|  | XGBoost | 0.7544 | (0.7302–0.7777) | 0.7298 | 0.932 | 0.50 / 0.0705 | 0.041 / 0.647 | 0.999 / 0.745 | 0.688 | 0.933 | 0.077 | 0.0579 |
|  | SVM | 0.5550 | (0.5267–0.5827) | 0.5439 | 0.931 | 0.50 / 0.0686 | 0.022 / 0.297 | 0.999 / 0.904 | 0.706 | 0.932 | 0.043 | 0.0623 |
|  | LightGBM | 0.7540 | (0.7301–0.7766) | 0.7182 | 0.933 | 0.50 / 0.0707 | 0.058 / 0.646 | 0.998 / 0.750 | 0.660 | 0.934 | 0.106 | 0.0582 |
|  | MLP | 0.6663 | (0.6407–0.6897) | 0.6421 | 0.931 | 0.50 / 0.0826 | 0.000 / 0.629 | 1.000 / 0.643 | 0.000 | 0.931 | 0.000 | 0.0638 |
| TyG-BMI panel | Logistic | 0.7511 | (0.7276–0.7751) | 0.7350 | 0.933 | 0.50 / 0.0768 | 0.039 / 0.614 | 0.999 / 0.784 | 0.778 | 0.933 | 0.074 | 0.0576 |
|  | DecisionTree | 0.7230 | (0.6988–0.7490) | 0.7033 | 0.930 | 0.50 / 0.0752 | 0.033 / 0.716 | 0.997 / 0.622 | 0.486 | 0.933 | 0.062 | 0.0588 |
|  | Lasso | 0.7513 | (0.7277–0.7752) | 0.7351 | 0.933 | 0.50 / 0.0768 | 0.039 / 0.614 | 0.999 / 0.784 | 0.778 | 0.933 | 0.074 | 0.0576 |
|  | Ridge | 0.7529 | (0.7296–0.7753) | 0.7349 | 0.933 | 0.50 / 0.0729 | 0.043 / 0.612 | 0.999 / 0.777 | 0.767 | 0.933 | 0.081 | 0.0575 |
|  | ENet | 0.7513 | (0.7277–0.7752) | 0.7351 | 0.933 | 0.50 / 0.0767 | 0.039 / 0.614 | 0.999 / 0.784 | 0.778 | 0.933 | 0.074 | 0.0576 |
|  | KNN | 0.7056 | (0.6795–0.7300) | 0.6912 | 0.931 | 0.50 / 0.0938 | 0.009 / 0.540 | 1.000 / 0.797 | 0.625 | 0.931 | 0.018 | 0.0598 |
|  | RandomForest | 0.7487 | (0.7249–0.7722) | 0.7342 | 0.932 | 0.50 / 0.0831 | 0.024 / 0.571 | 1.000 / 0.824 | 0.812 | 0.932 | 0.047 | 0.0582 |
|  | XGBoost | 0.7534 | (0.7298–0.7759) | 0.7342 | 0.932 | 0.50 / 0.0833 | 0.037 / 0.590 | 0.999 / 0.804 | 0.667 | 0.933 | 0.070 | 0.0580 |
|  | SVM | 0.5809 | (0.5525–0.6087) | 0.5684 | 0.931 | 0.50 / 0.0701 | 0.019 / 0.299 | 0.999 / 0.908 | 0.588 | 0.932 | 0.036 | 0.0629 |
|  | LightGBM | 0.7466 | (0.7238–0.7691) | 0.7220 | 0.932 | 0.50 / 0.0724 | 0.046 / 0.623 | 0.998 / 0.761 | 0.595 | 0.933 | 0.086 | 0.0586 |
|  | MLP | 0.6697 | (0.6436–0.6936) | 0.6871 | 0.931 | 0.50 / 0.0907 | 0.000 / 0.596 | 1.000 / 0.699 | 0.000 | 0.931 | 0.000 | 0.0630 |

AUC, area under the receiver-operating characteristic curve; CI, confidence interval; CV, cross-validation; NPV, negative predictive value; PPV, positive predictive value. Confidence intervals were obtained from 500 bootstrap resamples of the validation set. Nested CV AUC is the mean of five outer folds, with hyperparameters tuned in an inner three-fold loop; it is reported to document the absence of substantial optimism. Sensitivity and specificity are given at the default probability threshold of 0.50 and, after the slash, at the Youden-optimal threshold; the numeric value of that threshold is given for every model in the column headed "Threshold". Because the threshold was selected in the same validation set in which these metrics were then calculated, the accuracy, sensitivity, specificity, predictive value and F1 columns are exploratory, whereas AUC and the Brier score are threshold-independent. Because the outcome is imbalanced, the default threshold classifies almost all participants as unaffected, which is why sensitivity is very low at 0.50.

**Table S7. Sensitivity analysis including the participants outside the reference age range (full sample, aged 6–21 years).**

| **Exposure** | **Model** | **Full sample OR per 1 SD** | **Full sample Q4 vs Q1** | **Primary analysis (aged 7–17 years) OR per 1 SD** |
| --- | --- | --- | --- | --- |
| TyG | Model III | 1.333 (1.174–1.513) | 1.520 (1.232–1.876) | 1.298 (1.139–1.480) |
|  | Model IV | 1.347 (1.186–1.529) | 1.537 (1.245–1.897) | 1.306 (1.145–1.489) |
| TyG-BMI | Model III | 2.090 (1.970–2.216) | 3.834 (3.176–4.629) | 2.108 (1.985–2.238) |
|  | Model IV | 2.087 (1.967–2.215) | 3.878 (3.211–4.685) | 2.109 (1.985–2.241) |

The primary analysis was restricted to participants aged 7–17 years because the blood-pressure reference values used to define hypertension are tabulated only for this range. The full sample additionally includes the 2,026 participants (7.26%) who were younger than 7 years (656) or aged 18 years or older (1,370); in the full sample, hypertension was classified by the study centres and the diagnostic thresholds applied to these participants could not be verified from the de-identified dataset. The full sample comprised 27,912 participants (prevalence 6.76%), and the primary analysis 25,886 participants (prevalence 6.95%). Quartiles were recomputed within each analysis set.

**Table S8. Sequential attenuation analysis: addition of waist-to-height ratio and BMI to the fully adjusted model.**

| **Exposure** | **Model** | **OR per 1 SD (95% CI)** | **p value** |
| --- | --- | --- | --- |
| TyG | Model III (fully adjusted) | 1.298 (1.139–1.480) | < 0.001 |
|  | + waist-to-height ratio | 1.204 (1.053–1.378) | 0.007 |
|  | + waist-to-height ratio and BMI | 1.158 (1.011–1.326) | 0.034 |
| TyG-BMI | Model III (fully adjusted) | 2.108 (1.985–2.238) | < 0.001 |
|  | + waist-to-height ratio | 1.883 (1.657–2.138) | < 0.001 |
|  | + waist-to-height ratio and BMI | 1.914 (1.134–3.231) | 0.015 |

Estimates are odds ratios per 1 SD of the exposure in the primary analysis set (aged 7–17 years). This analysis is descriptive: because the design is cross-sectional, it does not estimate a causal mediation effect, and the attenuation observed indicates only that part of the association is shared with adiposity.

**Table S9. Cluster-sensitivity assessment based on the design effect.**

| **Exposure** | **Mean cluster size** | **ICC = 0.01** | **ICC = 0.02** | **ICC = 0.03** | **ICC = 0.05** |
| --- | --- | --- | --- | --- | --- |
| TyG | 30 | 1.12–1.52 | 1.11–1.54 | 1.09–1.56 | 1.06–1.60 |
|  | 50 | 1.11–1.53 | 1.09–1.57 | 1.06–1.61 | 1.02–1.67 |
|  | 100 | 1.08–1.57 | 1.04–1.64 | 1.00–1.70 | 0.95–1.80 (ns) |
|  | 200 | 1.04–1.64 | 0.97–1.75 (ns) | 0.92–1.85 (ns) | 0.85–2.02 (ns) |
|  | 400 | 0.97–1.75 (ns) | 0.88–1.94 (ns) | 0.81–2.10 (ns) | 0.72–2.38 (ns) |
| TyG-BMI | 30 | 1.97–2.26 | 1.95–2.28 | 1.94–2.29 | 1.92–2.32 |
|  | 50 | 1.96–2.27 | 1.94–2.30 | 1.92–2.32 | 1.88–2.36 |
|  | 100 | 1.94–2.30 | 1.90–2.34 | 1.87–2.38 | 1.82–2.45 |
|  | 200 | 1.90–2.34 | 1.84–2.41 | 1.80–2.48 | 1.73–2.58 |
|  | 400 | 1.84–2.41 | 1.76–2.53 | 1.70–2.62 | 1.60–2.78 |

School and city identifiers were not present in the de-identified dataset, so clustering could not be modelled directly. Instead, standard errors were inflated by √DE with DE = 1 + (m − 1)ρ, where m is the mean cluster size and ρ the intra-cluster correlation. Cells give the resulting 95% confidence interval for the odds ratio per 1 SD of the exposure (Model IV); "ns" marks combinations in which the association would no longer be statistically significant. The unadjusted odds ratio was 1.306 (95% CI 1.145–1.489) for TyG and 2.109 (1.985–2.241) for TyG-BMI; the association with TyG-BMI remained significant in all 20 scenarios, whereas the association with TyG remained significant in 12 of the 20 scenarios, up to a design effect of 3.97; solving for the design effect at which its two-sided p value would reach 0.05 gives DE* = 4.12, so the TyG association is robust to clustering unless the product of average cluster size and intra-cluster correlation is unusually large.

**Table S10. Incremental value of each index over the same baseline model, which already includes BMI.**

| **Model** | **n predictors** | **AUC** | **ΔAUC vs baseline** | **95% CI of ΔAUC** | **DeLong p** |
| --- | --- | --- | --- | --- | --- |
| Baseline | 6 | 0.7465 | — | — | — |
| Baseline + TyG | 7 | 0.7493 | +0.0028 | +0.0001 to +0.0055 | 0.040 |
| Baseline + TyG-BMI | 7 | 0.7486 | +0.0021 | -0.0001 to +0.0043 | 0.056 |
| Baseline + TyG + TyG-BMI | 8 | 0.7495 | +0.0030 | +0.0001 to +0.0059 | 0.040 |

One common baseline was constructed from the intersection of the features selected in the two panels after removing TyG and TyG-BMI themselves (BMI, CI, HDLC, HR, LDLC, UA). The same training/validation split and the same training-set-fitted k-nearest-neighbour imputation were used for all four models, so the only difference between them is which index was added. Logistic regression with standardisation was used throughout; the DeLong test for correlated curves compared each augmented model with the baseline in the same held-out validation set (n = 7,766). The increments are statistically detectable but very small (about 0.003 in AUC). AUC, area under the receiver-operating characteristic curve; CI, confidence interval.

**Table S11. Robustness of the reported models to the rule used to break ties in feature-selection frequency.**

| **Panel** | **Features with frequency ≥ 2** | **Retained** | **Predictors finally used** |
| --- | --- | --- | --- |
| TyG | 8 | 8 | BMI, CI, HR, LDLC, HDLC, UA, TyG, WHtR |
| TyG-BMI | 10 | 8 | CI, HR, LDLC, HDLC, UA, BMI, gender, education |

| **Analysis** | **Lowest AUC** | **Median AUC** | **Highest AUC** | **Reported set** |
| --- | --- | --- | --- | --- |
| All subsets of the tied pool (n = 36) | 0.7383 | 0.7507 | 0.7539 | 0.7513 |
| Subsets containing TyG-BMI (n = 28) | 0.7383 | 0.7507 | 0.7539 |  |
| Subsets not containing TyG-BMI (n = 8) | 0.7394 | 0.7502 | 0.7516 |  |
| XGBoost, predictors as reported |  | 0.7534 |  |  |
| XGBoost, education replaced by TyG-BMI |  | 0.7531 |  |  |
| XGBoost, TyG-BMI added as a ninth predictor |  | 0.7571 |  |  |

In the TyG panel exactly eight features reached a frequency of two or more, so the consensus rule was unambiguous and TyG itself was retained. In the TyG-BMI panel ten features reached that frequency while the rule retains eight, so two had to be dropped; ties were broken by the order in which features first appeared in the concatenated output of the three algorithms (Lasso, then Boruta, then SVM-RFE), which excluded waist-to-height ratio and TyG-BMI. The first three rows enumerate every alternative way of breaking that tie, evaluated with logistic regression on the same split and the same imputation; the last three rows re-evaluate three specific predictor sets with the same XGBoost specification used in Table S6. The choice among tied features has a negligible effect on discrimination, and including or excluding TyG-BMI does not by itself explain the performance achieved. AUC, area under the receiver-operating characteristic curve.
